# Robust out-of-distribution prediction of Buchwald–Hartwig reactions

**DOI:** 10.1038/s43588-026-01017-6

**Published:** 2026-08-10

**Authors:** Paulo Neves, Bo Hao, Santeri Aikonen, Justin B. Diccianni, Jörg K. Wegner, Philippe Schwaller, Iulia I. Strambeanu

**Affiliations:** 1 Drug Discovery Data Science, In Silico Discovery, Johnson & Johnson, Porto Salvo, Portugal; 2 Laboratory of Artificial Chemical Intelligence (LIAC), Institut des Sciences et Ingénierie Chimiques, Ecole Polytechnique Fédérale de Lausanne (EPFL), Lausanne, Switzerland; 3 Chemistry Capabilities, Analytical and Purification, Global Discovery Chemistry, Johnson & Johnson, Spring House, PA USA; 4 Drug Discovery Data Science, In Silico Discovery, Johnson & Johnson, Spring House, PA USA; 5 Drug Discovery Data Science, In Silico Discovery, Johnson & Johnson, Cambridge, MA USA; 6 National Centre of Competence in Research (NCCR) Catalysis, Ecole Polytechnique Fédérale de Lausanne (EPFL), Lausanne, Switzerland

**Keywords:** Catalysis, Computational science

## Abstract

The Buchwald–Hartwig cross-coupling is a cornerstone of modern pharmaceutical synthesis, yet predictive modeling of its outcomes remains constrained by data quality and chemical space coverage. Electronic laboratory notebooks contain heterogeneous, noisy records, while open-source high-throughput experimentation (HTE) datasets are fragmented and narrow in scope, leading to poor model performance on unseen substrates and conditions. Here we introduce a framework that systematically standardizes and integrates multiple reaction datasets into a high-quality, unique-structure-per-entity dataset, coupled with active learning to strategically expand chemical space. By merging published Buchwald–Hartwig HTE data with new experimental results, we achieve a model with predictive power across novel substrates and conditions, delivering improved out-of-distribution predictions compared with previous approaches. Crucially, model-guided reagent recommendations were validated experimentally, confirming the framework’s utility to uncover unexplored reactivity. This work establishes a blueprint for robust machine learning in synthetic chemistry and enables preemptive in silico reagent screening to accelerate pharmaceutical discovery.

## Abstract (web summary)

A predictive model capable of generalizing to previously unseen Buchwald–Hartwig chemical space is built to enable rapid, in silico optimization, reducing reaction scouting time from weeks to minutes.

## Main

The Buchwald–Hartwig (BH) cross-coupling reaction^1,2^ has become an indispensable tool in modern synthetic chemistry^3^, particularly in the pharmaceutical industry, where it is used to synthesize structurally diverse (hetero)aryl amine compounds^4^. The ability to reliably forecast the outcomes of BH reactions is thus invaluable, with machine learning (ML) methods offering a pathway to streamline and accelerate lead discovery efforts^5,6^. A recent example introduced a tool^7^ to estimate substrate-adaptive conditions for palladium-catalyzed C–N couplings, trained on a diverse experimental dataset of reactant pairings and systematically refined through active learning. Beyond predicting reaction outcomes^8–11^, the integration of reagent dictionaries^12^ enables yield prediction models to suggest the most suitable catalysts, bases, solvents and other conditions to achieve desired transformations. Retrosynthesis planning^13–15^, which identifies viable synthetic routes to target molecules, can further benefit from accurate reaction-level predictors, as robust models of cross-coupling chemistry directly expand the reliability of multistep route design. However, the inherent complexity and sensitivity to conditions in these reactions pose substantial challenges for traditional predictive models^16–18^. Addressing these complexities requires not only vast numbers of data but also a degree of consistency and precision in data generation that is often difficult to achieve, in part due to the heterogeneity of the data sources.

Mining data from electronic laboratory notebooks (ELNs) is one possible avenue towards building predictive synthesis models, but the performance of these models is capped due to data quality. ELN and literature scraped datasets^19^ can vary widely in reaction set-up, purification processes and yield reporting. Additionally, such datasets have heterogeneous, and sometimes contradictory, representations of the same chemical entities, do not capture all reaction components in a tabular format and, in the case of literature, lack negative examples.

While better algorithms are still required to tackle this industry-wide challenge^17^, high-throughput experimentation (HTE) has emerged as a powerful method for generating reaction data with high consistency and quality through controlled and automated processes. Recently, a large-scale release of over 39,000 previously proprietary HTE reactions has further enriched the chemical data landscape^20^, enabling systematic exploration, which unveiled hidden statistical relationships between reaction components and uncovered dataset biases.

Similarly, parallel library synthesis has been instrumental in drug discovery, enabling the efficient production of structurally diverse analogs through robust synthetic methodologies^21^. Dombrowski et al.^22^ analyzed over 5,000 parallel libraries across 14 years, showcasing the evolution and proven success of these approaches. Notably, BH cross-coupling reactions were identified as one of the most challenging, with success rates among the lowest. Nonetheless, HTE platforms have demonstrated notable success in optimizing BH cross-coupling reactions, as reported in ref. ^23^, offering robust tools for synthesizing complex molecules on micro- and nanomolar scales and under automation-friendly conditions.

Collectively, these advancements established a foundation for reliable and standardized data generation, essential for navigating the inherent complexities of predictive BH reaction modeling. Moreover, HTE allows for the quick accumulation of a vast number of data points tailored to specific needs, encompassing a wide range of substrates and diverse reaction conditions. The ability to efficiently explore diverse reaction environments and generate comprehensive data makes HTE an ideal source of data for ML models aimed at estimating reaction outcomes, particularly when robust out-of-distribution (OOD) performance is required.

ML models excel at identifying complex patterns within large datasets^24^, and when trained on high-quality data, such as HTE data, they can more accurately predict reaction outcomes, even for previously unseen substrates. This is the reason why in recent years there has been an increase in studies combining ML and HTE^7,25–27^. The integration of HTE and ML therefore has the potential to transform reaction prediction by delivering models capable of OOD generalization—where predictions remain reliable despite differences between training and test data. This synergy is particularly impactful for applications in synthetic and medicinal chemistry, where the ability to forecast reaction outcomes in new, untested scenarios can substantially improve the time and resource efficiency required for chemical exploration and development.

**Fig. 1:**
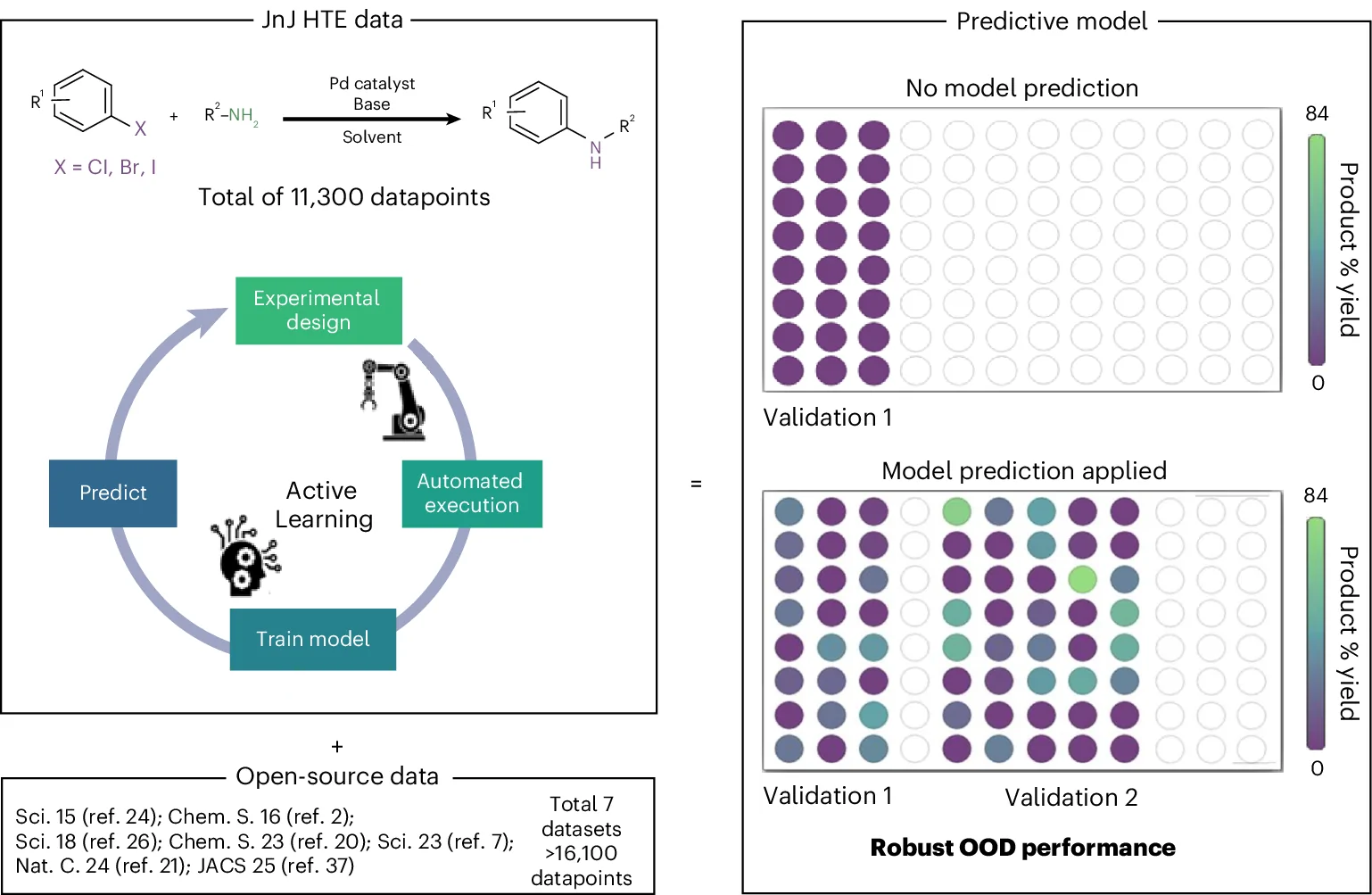
High-level project overview. A model with robust predictive performance is obtained through the combination of internally generated datasets and open-source HTE data. All open-source datasets used in this study are listed with an abbreviated journal name and publication year, and their corresponding reference number. By applying this model, previously unsuccessful reactant pairs were rescued, and challenging, OOD substrates were successfully coupled. JnJ, Johnson & Johnson.

The goal of this study is to develop a predictive model with wide OOD capabilities for BH reactions by integrating a newly generated HTE dataset with available open-source HTE data (Fig. 1). To this end, we employed a data-driven active learning^28^ approach that iteratively refined the model’s accuracy and generalization capacity across diverse chemical spaces. By standardizing and unifying disparate datasets into a single, high-quality format, we have created a comprehensive dataset that supports a model capable of reliable predictions across a meaningful area of the BH reaction landscape. This work not only advances the state of predictive chemistry for a critical reaction type but also demonstrates the potential of HTE and ML integration as a general framework for achieving robust, adaptable reaction prediction models and reagent-recommender systems.

## Results

**Fig. 2:**
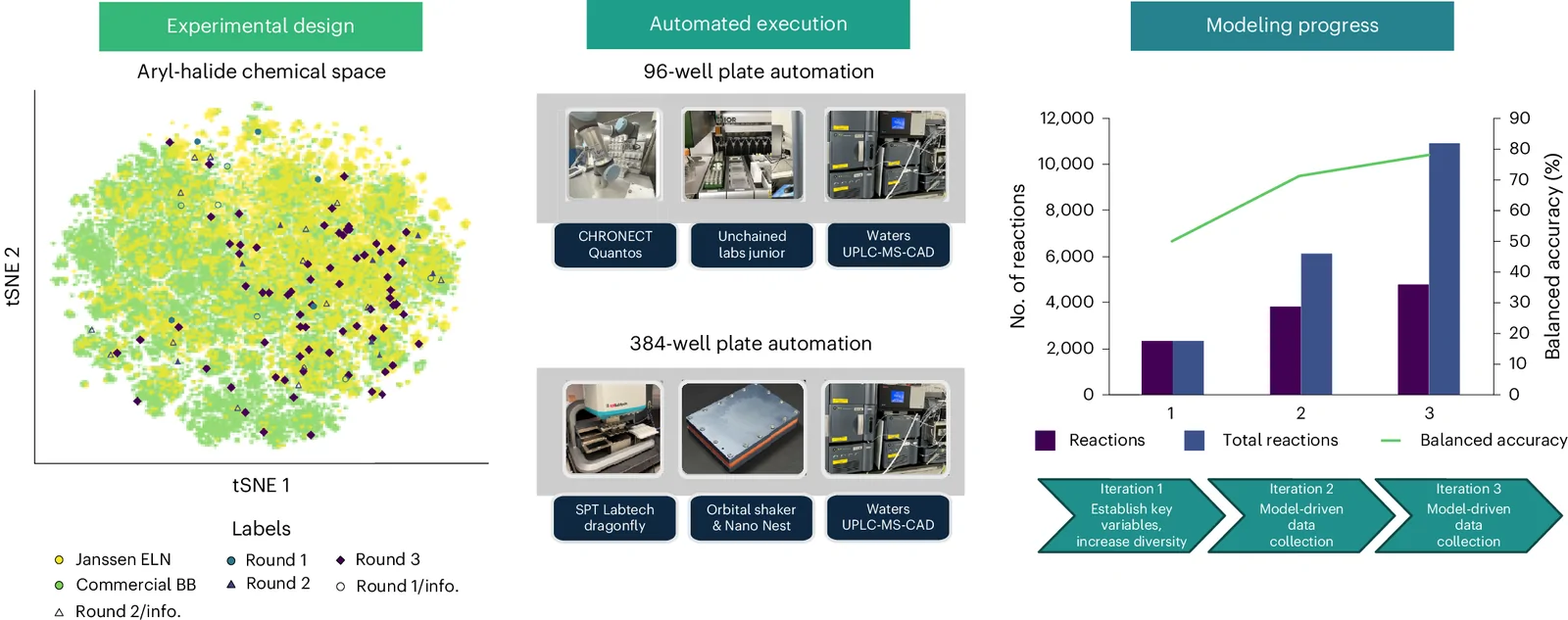
Automation- and HTE-fueled model build. Left: chemical-space coverage and overlap of data-production compounds with ELN entries. tSNE, *t*-distributed stochastic neighbor embedding. Middle: automated platforms utilized in the execution of HTE campaigns. Right: continued improvements in model quality were observed under the active learning approach, as indicated by the balanced accuracy. info., informer set. Source data

In this project, we strategically leverage automated workflows using 96-well and 384-well plates to execute our HTE designs, with product quantification performed using ultraperformance liquid chromatography (UPLC)^29^-MS-CAD (mass spectrometry–charged aerosol detector)^30^ (Fig. 2). We employed an iterative approach for data generation, purposefully selecting experimental conditions across three iterations. The first iteration focused on establishing key variables and increasing diversity in reaction conditions by exploring combinations of catalysts, bases and solvents. For iterations 2 and 3, we employed model-guided experimental design, targeting broader coverage of the substrate chemical space. Reactions with high model uncertainty were prioritized, enabling continuous refinement of the predictive model’s accuracy on the broader space. This systematic approach substantially enhanced data diversity and quality, resulting in the largest and most comprehensive BH HTE dataset in literature to date, with 11,300 (11.3k) high-quality reactions. The production of these data followed a quantitative methodology consisting of two large phases. The first phase aimed to produce a starting baseline ML model, which was used in a second phase to perform experimental design in an active learning formulation. Additionally, we curated and standardized all other published BH HTE datasets into a unified format, creating an extensive dataset with ~27.5k reactions. To ensure comparability across heterogeneous quantification methodologies (for example CAD, isolated yield, MisER and so on), we harmonized all reactions under a binary classification framework using a 10% yield threshold. This cutoff reflects a standard go/no-go criterion in medicinal chemistry and permits consistent integration of multisource data.

The strategy for the first phase was centered around four factors: (1) informer set, (2) structure diversity criteria, (3) space density criteria and (4) undesired substructure filtering (Supplementary Section 2).

The second phase embraced an active learning methodology utilizing (1) BERT Enriched Embedding^12^, an extension of a language model for yield prediction^16^, which was trained on the data from the previous phase, (2) exploration of a virtual BH space of 64,000,000 (64M) reactions, (3) the Ranked Batch-Mode Active Learning^31^ function using model uncertainty and Tanimoto distance from previous selections and (4) diversity and density (Supplementary Section 3).

We address model bias in benchmarking by training six ML models with two types of features, the differential reaction fingerprint^32^ (DRFP) and a vector of physicochemical attributes, and by training one artificial intelligence Simplified Molecular Input Line Entry System (SMILES)-based model. We address data bias^33^ by testing on OOD data; OOD was created by clustering all available reactions with DRFP and *k*-means^34^ and only testing in the clusters where source is not represented. Finally, we addressed metric bias^35^ by considering a total of eight performance metrics and identifying four that can summarize the capabilities of interest (uncertainty estimation, good precision and serviceable recall in class imbalance OOD scenarios) of the different models.

**Fig. 3:**
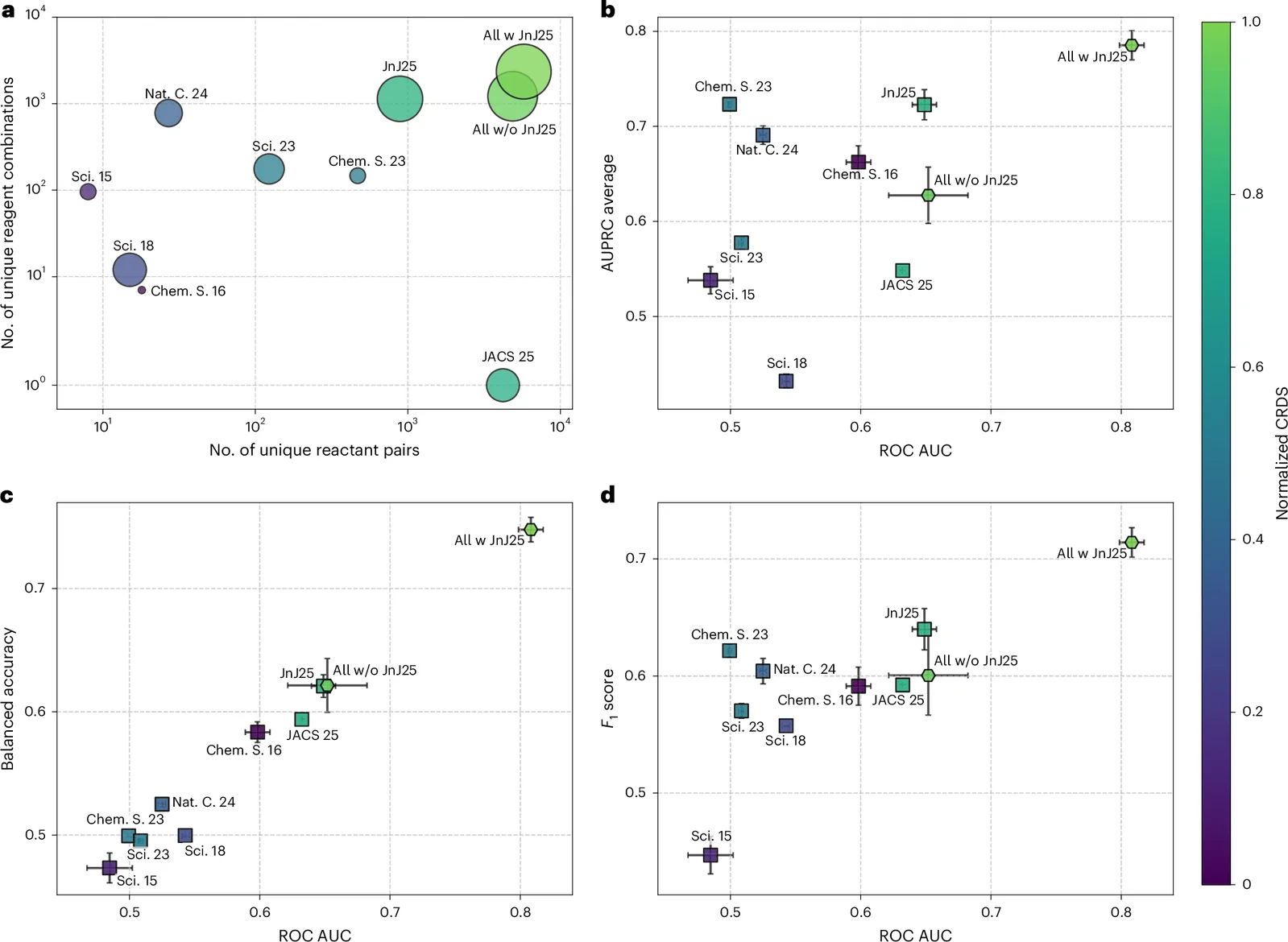
Evaluation of model metrics on the basis of data source. **a**, Number of unique substrate pairs against number of unique reagent combinations. Each of the eight open-source BH HTE datasets used in the project is represented in size; these are colored according to CRDS (a data-centric score with strong correlation to OOD performance). ‘All w/o JnJ25’ denotes the combined open-source Buchwald–Hartwig HTE datasets excluding the JnJ25 dataset. **b**–**d**, Performance across four critical metrics for the best ML model for each given dataset when tested on OOD clusters built from all remaining open-source data. Each square marker represents the mean performance across *n* = 10 independent random cluster initializations, where each initialization uses a different random seed for *k*-means clustering (*k* = 12 clusters). Error bars indicate s.d. across the ten cluster initializations. For each data source, substrates present in the training set were excluded from test clusters, creating a strict OOD evaluation. Hexagon markers represent the combined All w JnJ25 dataset, evaluated across *n* = 10 iterations where in each iteration four cycles of train–test splits (three clusters held out, nine used for training) were performed until all 12 clusters appeared in the test set once, with performance averaged first across the four cycles within each iteration, then across all ten iterations. **b**–**d**, AUPRC reflects how effectively the model enriches for successful reactions when ranking candidates (all thresholds) (**b**); balanced accuracy reflects how effectively the model makes correct predictions for both successful and unsuccessful reactions, independent of class imbalance (**c**); *F*_1_ score reflects how effectively the model converts predictions into correct operational go/no-go decisions at a chosen threshold (**d**). AUPRC, area under the precision–recall curve. Source data

The data produced in this study, labeled JnJ25 in Fig. 3, surpass the previous studies in our comparison not only in overall size but also in diversity of reagent combinations, with the second highest diversity of unique substrate pairs, behind a recently published work^36^ (Fig. 3a). The combination of JnJ25 data with all remaining BH HTE open-source datasets is denominated ‘All w JnJ25’.

Unexpectedly, we discovered that model performance on OOD predictions was determined not primarily by dataset size, but rather by a previously unrecognized relationship between data diversity and prediction accuracy, as shown in Table 1. This finding challenges the conventional wisdom that larger datasets necessarily lead to better generalization, instead showing that strategic diversity in reaction space coverage is the key driver of robust predictions. We introduce a new data-centric metric, Compound-Reaction Diversity Score (CRDS), which aims to estimate the ability of potential models trained on a given dataset to perform in OOD scenarios. This metric can be employed retrospectively to elucidate the key dataset attributes that underpin the effective training of ML models for robust OOD predictions, as well as in prospective planning of a data production campaign.

**Table 1:** Pearson and Spearman correlations between CRDS, simplified CRDS and size of the training dataset against ML model performance on synthesis success prediction of reactions in OOD clusters

	Pearson				Spearman			
	ROC AUC	Bal. acc.	*F*_1_ score	AUPRC	ROC AUC	Bal. acc.	*F*_1_ score	AUPRC
CRDS	**0.790**	**0.770**	**0.308**	0.170	0.452	**0.429**	0.310	0.286
Simplified CRDS	0.534	0.558	0.263	**0.381**	0.262	0.214	**0.429**	**0.405**
Dataset size	0.603	0.541	0.287	0.062	**0.476**	0.405	−0.214	−0.310

Bold values indicate the highest correlation for each metric when comparing three methods. Bal. acc., balanced accuracy.

Its formula combines the number of unique reactant pairs, unique reagent combinations and the size of the dataset. Additionally, each component has adjustable thresholds to induce diminishing returns and varying weights. When applying this calculation, we found a 0.79 Pearson correlation between CRDS and receiver operating characteristic-area under the curve (ROC AUC) in the most challenging OOD test, indicating a high degree of correlation between substrate diversity quantity and OOD performance (Table 1). More details of the CRDS formula and calculation can be found in Supplementary Section 4.

Figure 3b–d presents the results of two benchmarking experiments. In these panels, the square symbols represent the highest-performing combinations of ML model, feature vector and data source, evaluated on OOD clusters in which the corresponding data source is not represented. Furthermore, any test instances containing a substrate present in the training set were excluded. This creates a very challenging OOD test, as the DRFP contained reagents, leading to cluster formation impacted by both reagent and substrate scope. The objective of this analysis was not to maximize predictive performance, but rather to compare the relative utility of each data source in enabling the training of models that are effective for reagent screening and experimental design in previously unexplored BH chemical spaces.

Hexagon symbols quantify the performance gain obtained by incorporating the JnJ25 dataset into all previously available open-source BH HTE datasets. This evaluation was conducted using the DRFP OOD cluster test, in which reactions—represented as DRFPs incorporating both reagents and substrates—were partitioned into 12 clusters via *k*-means clustering. The test procedure comprised ten iterations: in each iteration, there is a cycle where three clusters were selected without replacement as the test set, while the remaining nine were used for training; the cycle progresses until all clusters have appeared in the test set once. Performance was first averaged across the cycle with the held-out clusters within each iteration, and then across all iterations to determine the metrics value represented by the hexagon in the scatter plot. We also demonstrate how a binary reactivity classification model can be used as a reagent recommender and HTE plate designer (recommending both substrates and reagents) when combining it with a reagent dictionary and uncertainty estimation. The reagent-recommender workflow using a reagent dictionary, feature construction and scoring pipeline is provided as a self-contained notebook in the repository (Supplementary Section 5).

**Fig. 4:**
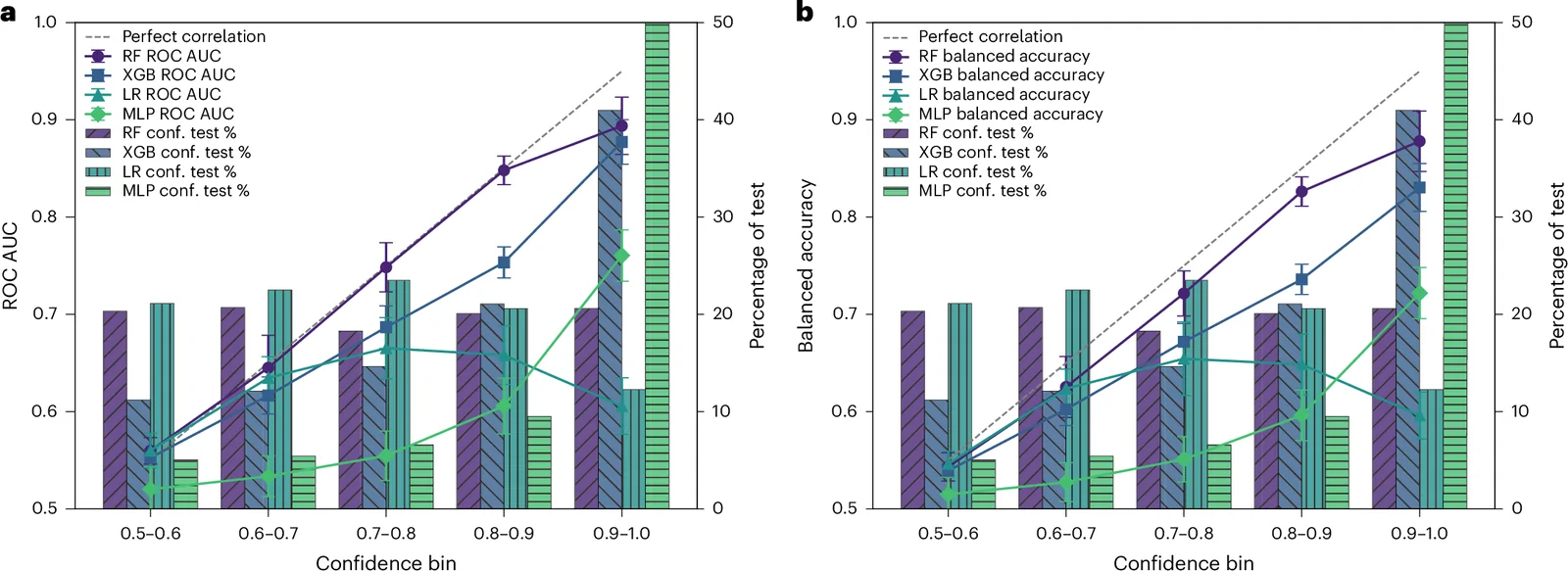
Model uncertainty calibration and confidence-binned performance for OOD predictions. **a**,**b**, Calibration curves showing the relationship between model confidence (*x* axis) and ROC AUC (**a**) (right *y* axis) or balanced accuracy (**b**) (left *y* axis) across five confidence bins for OOD test reactions (*n* = 20 independent random cluster initializations). The right *y* axis shows the percentage of test data predicted within the respective confidence interval. Each point represents the mean performance within a confidence bin, with error bars indicating s.d. across the 20 cluster initializations. The gray dashed line represents perfect calibration where confidence is equal to success rate. Confidence values are derived from model prediction probabilities, inversely related to uncertainty and normalized between 0.5 and 1. LR, logistic regression; conf., confidence. Source data

Before utilizing these types of models in the laboratory or executing experimental validation, it is important to assess whether the models are uncertainty calibrated, as uncertainty will be used to rank reagent and substrate pair combinations. We tackle this by binning the predictions of OOD reactions into five confidence bins. The confidence value is inversely derived from uncertainty and normalized between 0.5 and 1 (Supplementary Section 4.3, Supplementary equation (1)) as described in ref. ^12^. If the model is perfectly calibrated then performance will perfectly correlate with confidence, following the gray dashed line in Fig. 4.

Through the use of the confidence metrics, it is possible to rank reagent combinations for any given substrate pair by their likelihood of success. Furthermore, we found that a relatively simple random forest (RF)^37^ model, when trained on our properly curated dataset, achieved >90% ROC AUC on high-confidence OOD predictions, demonstrating that model architecture sophistication may be less critical than previously thought, at least for achieving reliable chemical prediction in a single reaction type with chemical space defined in ~27,500 samples.

To further investigate this observation, we performed hyperparameter optimization for the three top-performing model families (RF, histogram-based gradient boosting (XGB) and multilayer perceptron (MLP)) across two feature representations: full-reaction DRFP and a split representation separating substrate–product DRFP from reagent physicochemical descriptors (Supplementary Section 5). The results revealed that the choice of feature representation had a larger impact on OOD performance than the choice among top ML models. This suggests that, when the feature representation cleanly encodes the relevant chemical information, separating substrate structural identity from reagent properties, a simple model that does not distort it is sufficient. RF is particularly well suited to this task because tree-based splits on binary fingerprint bits directly correspond to ‘substructure present/absent’ decisions, and the ensemble’s natural subsampling prevents overfitting to training-distribution artifacts. We expect pretrained transformers to become more advantageous as additional reaction types and larger chemical spaces are incorporated. A detailed discussion of the model selection rationale is provided in Supplementary Section 5. It is also worth noting that richer descriptor families such as quantum-mechanics-informed features enriching deep learning embeddings represent promising directions for future work.

### Experimental validation results

**Fig. 5:**
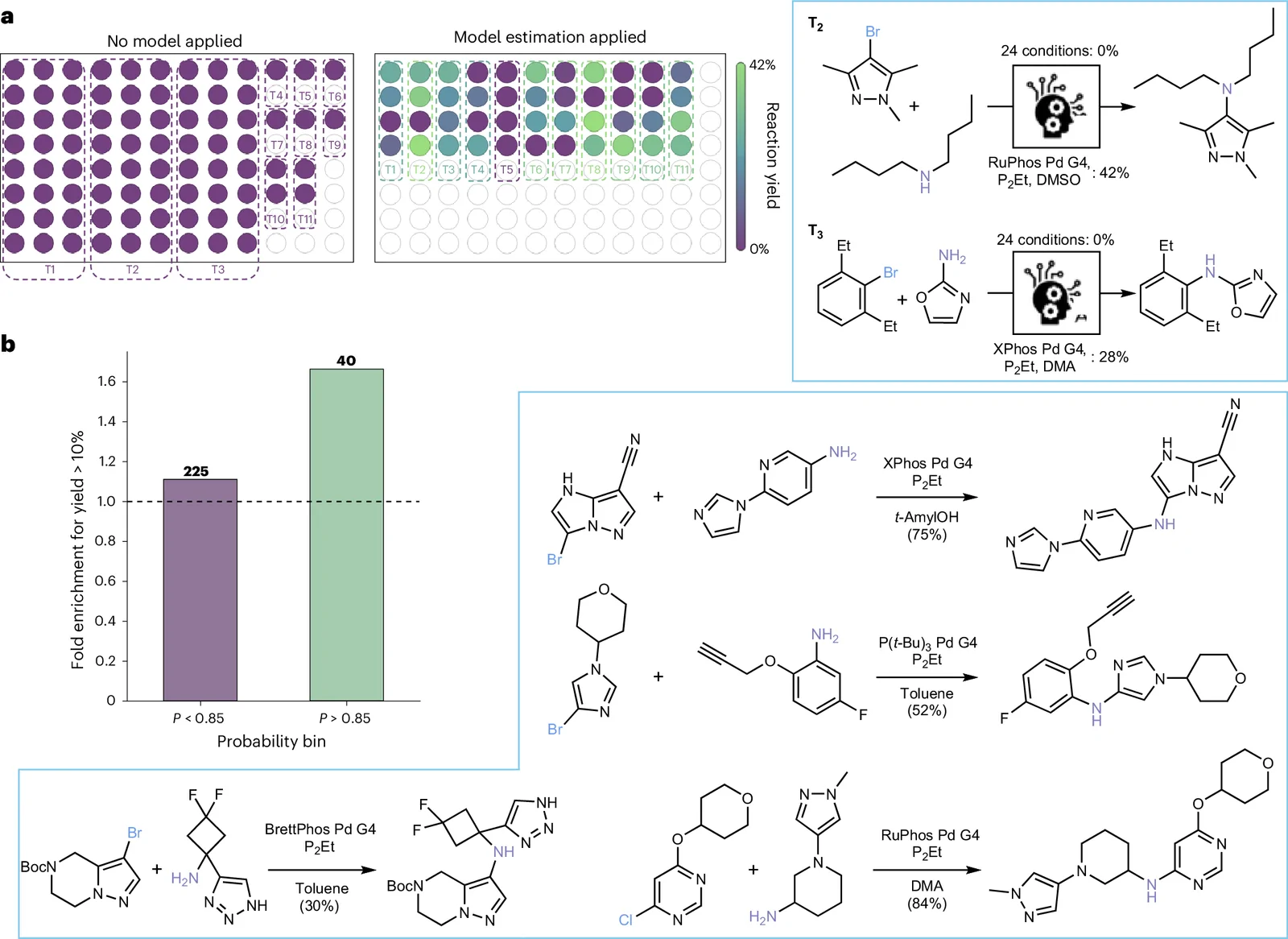
Experimental validation. **a**, 11 previously unsuccessful aryl–amine pairs were tested across the four highest-ranked predicted reaction conditions, achieving viable synthetic routes for 10 of the 11 substrate pairs. **b**, Prospective validation of the model across a novel chemical reaction space; experimental results present fold enrichment of reactions selected from a vast chemical domain (~2.46 × 10^11^ reactions) predicted by the model with moderate confidence (<0.85) and high confidence (>0.85) versus a baseline set. The dashed horizontal line indicates the no-enrichment baseline. DMA, dimethylacetamide; T_*x*_, transformation *x*. Source data

The first experiment was aimed at rescuing historically unsuccessful substrates (Fig. 5a). In this scenario, we tested the practical applicability of our model by using it as a reagent recommender^12^ to identify successful reaction conditions for 11 historically unsuccessful aryl halide–amine substrate combinations gathered from HTE literature reactions as well as data produced during this project. Previously, three substrate pairs had each failed across 24 reaction conditions, two pairs had each failed across two reaction conditions and the remaining six pairs had each failed in one reaction condition. We then selected catalysts, bases and solvents that were frequently covered in the dataset (minimum 400 samples), leading to a total of 1,092 reagent condition combinations. Leveraging the model, we systematically ranked all these combinations according to predicted confidence. For each substrate pair, only the top four recommendations were experimentally tested. Out of these 44 reactions, 27 succeeded (yield > 10%), efficiently providing viable synthetic routes for 10 of the 11 substrates (91%). This illustrates the model’s ability to efficiently identify effective reaction conditions, substantially reducing the experimental burden and conserving laboratory resources.

The second experiment (Fig. 5b) was a prospective validation across a differentiated chemical space from the training data. We assessed the model’s predictive generalizability in a vast, unexplored chemical domain sampling from a virtual space consisting of 13 catalysts, 12 bases, 7 solvents, 12,252 aryl halides and 18,364 amines, representing approximately 2.46 × 10^11^ possible BH reactions. We specifically selected entirely novel aryl and amine combinations (absent from any previous training data) to rigorously test the model’s ability to identify successful reactions. From this space, we experimentally evaluated three groups of reactions.

- A baseline group of 4,800 reactions, selected via diversity, space density, pharmacological relevance and model uncertainty, which yielded around 19% success rate.
- Reactions predicted with moderate confidence (<0.85; for reference 0.5 indicates complete uncertainty) achieved a success rate of 21%, a fold enrichment of 1.11 over the baseline (Fig. 5b).
- Reactions predicted with high confidence (>0.85) achieved a success rate of 33%, representing a fold enrichment of 1.64 over the baseline (Fig. 5b).

Critically, these results highlight the model’s exceptional capacity to distinguish reactions where modeling can enrich from those out of domain, even when dealing with entirely novel chemical combinations drawn from an extraordinarily expansive chemical space.

Collectively, these experiments validate that our unified, actively curated ML model substantially enhances BH reaction predictions by (1) effectively rescuing historically unsuccessful reaction pairs through minimal additional experimentation and (2) confidently identifying viable reactions in previously unexplored chemical space. This approach offers a powerful, transferable framework for accelerating the discovery and optimization of pharmaceutically relevant chemical transformations, and the growing high-quality curated dataset enables further modeling research.

## Discussion

We have developed a unified, actively curated ML framework capable of robust OOD prediction for BH cross-coupling reactions. By systematically standardizing heterogeneous datasets, strategically expanding reaction space through active learning and validating predictions experimentally, we demonstrate that data diversity, rather than dataset size or model complexity alone, is the key driver of generalizable performance. This insight not only advances predictive chemistry for a pivotal reaction class but also establishes principles broadly applicable to other transformations.

The models developed in this project operate within the reagent space covered during training (32 palladium precatalysts, 12 bases, 12 solvents), which, while comprehensive for most experimental objectives, represents a subset of commercially available options. Predictions for entirely novel reagent classes not represented in training should be interpreted accordingly. The binary classification framework (10% yield threshold) reflects practical go/no-go decisions in medicinal chemistry but does not capture quantitative yield information. Notably, heterogeneous yield quantification methods across aggregated datasets (CAD, isolated yield, MisER, liquid chromatography area percent or LCAP) were harmonized through this binary threshold, with only 11.9% of reactions falling in the 5–15% boundary region where measurement variability could influence classification. The CRDS shows strong correlation with OOD performance for BH chemistry (Pearson *r* = 0.79 versus *r* = 0.60 for dataset size alone), but the weighting exponents were empirically fitted to this reaction class and would require recalibration for other transformations where combinatorial complexity differs.

An opportunity for future work remains the harmonization of noisy, heterogeneous reaction data (for example, industry ELNs), inclusion of conditions in a global setting and scaling predictive frameworks across reaction types. Better algorithms are still required to tackle these industry-wide challenges; potentially in the future the use of knowledgeable chemistry artificial intelligence agents^38,39^ will make possible a harmonization of the free-text protocol and tabular data, while noise detection techniques will be able to remove the most ‘obvious’ noisy samples from the training dataset^40–43^. Looking ahead, the integration of additional HTE campaigns and richer reaction context representations can further enhance predictive power.

## Methods

### HTE

#### Materials

Standard glovebox techniques were utilized for the handling of air- and moisture-sensitive reagents. All reagents and starting materials were procured from commercial suppliers and employed as received without further purification. Solvents were sourced from sealed anhydrous containers to ensure minimal exposure to atmospheric moisture. Catalysts were obtained from commercial suppliers and stored within an MBraun glovebox under an inert atmosphere.

1-ml clear glass shell vials (8 mm × 30 mm, Analytical Sales Services) and 96-position reactor blocks were used for 96-well-plate reactions. For 384-well-plate reactions, Axygen 384-well clear V-bottom 120-µL polypropylene deep-well plates were employed.

Appropriate safety precautions were taken when handling air- and moisture-sensitive reagents, as well as strong acids and bases.

#### Instruments

A CHRONECT Quantos was used to dispense solid materials. Tecan Fluent, Unchained Lab Junior, SPT Labtech dragonfly and Apricot PP5 were used as liquid handlers for different purposes. An IKA MATRIX Orbital Delta+ shaker was used for shaking and heating the 384-well plate in a Nano Nest (Analytical Sales & Services). UPLC-MS analysis was performed using a Waters ACQUITY system equipped with as SQD2 detector and a CAD.

#### Reaction in 96-well plate

The HTE was designed using Katalyst software, which facilitated the generation of automation execution files for both solid-dispensing and liquid-handler robots. All palladium Buchwald precatalysts (1 mmol, 10 mol%) and solid bases (12 mmol, 1.2 equiv.) were accurately dispensed into 1-ml vials arranged in a 96-well plate, using a CHRONECT Quantos system within a glovebox environment. Subsequently, aryl halide (10 mmol, 1.0 equiv., 50 ml of a 0.2-M stock solution) and amine (12 mmol, 1.2 equiv., 50 ml of a 0.24-M stock solution) were introduced via an Unchained Lab Junior liquid-handling system. Finally, the liquid bases (12 mmol, 1.2 equiv.) were directly added to the corresponding vials. The resulting reaction mixtures were sealed and stirred at 100 °C on an Unchained Lab Junior deck within the glovebox for a duration of 16 h.

#### Reaction in 384-well plate (liquid bases only)

The HTE was designed utilizing Katalyst software. Aryl halide (2 mmol, 1.0 equiv., 10 ml of a 0.2-M stock solution in 1,2-dichloroethane) and amine (2.4 mmol, 1.2 equiv., 10 ml of a 0.24-M stock solution in 1,2-dichloroethane) were dispensed into the corresponding wells of a 384-well plate using a Tecan Fluent liquid-handling system. Following this, the plates were subjected to a drying process to remove the solvent using a Genevac. Subsequently, the plates were transferred into a glovebox, where 10 µl of palladium Buchwald precatalysts (0.2 mmol, 10 mol%) and liquid bases (2.4 mmol, 1.2 equiv.), each dissolved in their respective reaction solvents, were added using an SPT dragonfly liquid dispenser. This ensured that the total volume of solvent for each reaction was 20 ml. After the addition of the reagents, the plates were sealed with Agilent PlateLoc and placed in a Nano Nest. The Nano Nest was maintained at a temperature of 100 °C and agitated at 800 r.p.m. on an orbital shaker for a duration of 16 h.

#### Reaction analysis

Following the reactions, analytical plates were prepared using an SPT Apricot PP5 system. For the 96-well-plate HTE, the reaction mixtures were diluted with 400 ml of dimethylsulfoxide (DMSO) and mixed. Subsequently, 50 ml of the resulting solution was transferred to a 96-well-plate analytical block containing 450 ml of CH_3_CN for further analysis. For the 384-well plates, the reaction mixtures were diluted with 40 ml of DMSO and mixed. From this solution, 2.5-ml aliquots were transferred into another 384-well plate containing 47.5 ml of CH_3_CN. The resulting analytical plates were then centrifuged and analyzed using UPLC-MS/CAD.

Calibration samples for CAD analysis were prepared using noscapine as the universal internal standard. The noscapine calibration samples were solutions in DMSO at the following concentrations: 1.024 mg ml^−1^, 0.512 mg ml^−1^, 0.256 mg ml^−1^, 0.128 mg ml^−1^, 0.064 mg ml^−1^, 0.032 mg ml^−1^, 0.016 mg ml^−1^ and 0.008 mg ml^−1^. Each of these calibration samples, along with the reaction samples, were analyzed using UPLC-MS/CAD in duplicate.

##### UPLC method

Solvent A, 0.1% trifluoroacetic acid in Milli-Q water;

Solvent B, 0.1% trifluoroacetic acid in Optima grade acetonitrile;

Gradient, 95% A/B to 0% A/B over 1.6 min, hold 0.4 min at 95% B;

Stop time, 2.3 min;

Flow rate, 2 ml min^−1^;

Column, CSH C18, 2.1 mm × 50 mm.

Data processing was carried out using Virscidian software. The calibration curve, represented by a quadratic equation, was drawn on the basis of the CAD peak areas of the noscapine calibration samples. The amount of product in the analytical samples was calculated using the CAD peak area corresponding to the products in the experimental samples, in conjunction with the established calibration curve. Product yields were determined on the basis of calibrated UPLC-MS/CAD peak areas using external standard curves and are reported as assay yields.

#### Comparative analysis of 96-well-plate workflow and 384-well-plate workflow

In the validation process, 6 substrate pairs and 64 reaction conditions were employed. The reactions within the 384-well-plate format were executed according to the layout depicted below, following the procedure outlined earlier. Subsequently, we compared these results with those obtained from the 96-well plate, also following the previously described procedure. To facilitate easier comparison, the results from the 96-well plate were reformatted to align with the 384-well layout. In conclusion, out of the 384 reactions, 228 exhibited differences of less than 5% when comparing the data from the 384-well plate and the 96-well plate. Additionally, 105 reactions showed differences ranging from 5% to 10%, while 51 reactions displayed differences between 10% and 13.2%. No reactions exhibited differences greater than 13.2%.

### Statistics and reproducibility

For the experimental data generation via HTE, no statistical method was used to predetermine sample size. Experimental data were generated using a model-guided selection strategy, where reactions were chosen on the basis of prediction uncertainty. No data were excluded from the analyses. The experiments were not randomized or blinded. Regarding statistical analysis and reproducibility, the model performance was evaluated using ten independent train–test splits with different random seeds (random_state = 0–9) for the data-source benchmark, creating distinct OOD cluster assignments via *k*-means clustering. Each train–test configuration enforced substrate-level OOD by excluding test reactions with aryl halides or amines present in training data. Performance metrics (balanced accuracy, ROC AUC, *F*_1_ score, AUPRC, precision, recall) were computed for each split, and mean ± s.d. were reported across all runs.

For the JnJ data impact analysis, models were trained with and without industrial HTE data across ten random seeds, with results aggregated to assess performance differences. For CRDS correlation analysis, Pearson and Spearman correlation coefficients were computed between diversity scores and OOD performance metrics across data sources.

Model training used fixed hyperparameters with consistent random seeds (random_state = 42 where applicable) to ensure reproducibility. All code, processed data and analysis notebooks are publicly available to enable full reproduction of results.

Regarding experimental reproducibility, for the HTE experimental validation, 384 reactions were performed in both 96-well and 384-well plate formats across 6 substrate pairs and 64 reaction conditions. Yield quantification via CAD showed that 228/384 reactions (59%) differed by <5%, 105/384 (27%) differed by 5–10% and 51/384 (13%) differed by 10–13.2% between formats, with no differences exceeding 13.2%, demonstrating reproducible quantification across experimental platforms.

## Supplementary information

Supplementary Information Supplementary Sections 1–5, Figs. 1–9 and Tables 1–8.

## Source data

Source Data Fig. 2 Statistical source data for bar charts.

Source Data Fig. 3 Statistical source data: dataset metrics and CRDS.

Source Data Fig. 4 Statistical source data: model calibration data.

Source Data Fig. 5 Statistical source data: experimental validation.

## Data Availability

The data for this project are available via GitHub at https://github.com/schwallergroup/bh-hte-ood and via Zenodo at https://doi.org/10.5281/zenodo.19636649 (ref. ^44^) a pickled version of the data with additional columns Source aggregated data with BH HTE datasets from which all figures are derived are available with this paper in the aforementioned GitHub and Zenodo. Specific tables with in silico results and datasets produced during experimental validation are also available in .zip format for Figs. 2–5. Source data are provided with this paper.

## Code availability

The code for this project is available via GitHub at https://github.com/schwallergroup/bh-hte-ood. A static version of the code repository can be found in Zenodo at https://doi.org/10.5281/zenodo.19635488 (ref. ^45^).
