# Supplementary material for: Robust out-of-distribution prediction of Buchwald–Hartwig reactions
Source: Nat Comput Sci. 2026 Aug 10;6(9):1015–23. doi: 10.1038/s43588-026-01017-6 (PMC13593570; doi:10.1038/s43588-026-01017-6)
Supplement: Supplementary file 1 [file 43588_2026_1017_MOESM1_ESM.pdf]

nature computational science

Supplementary information

https://doi.org/10.1038/s43588-026-01017-6

Robust out-of-distribution prediction of Buchwald–Hartwig reactions

In the format provided by the authors and unedited

Supplementary Information

Contents

1 Supplementary Section 1 - Data Generation via HTE **2**
1.1 Comparative Analysis of 96wp workflow and 384wp workflow 2

2 Supplementary Section 2 - Seed Data Selection and Production **4**
2.1 Initial Data Production and Baseline Model 4
2.2 First Selection and Data Generation 4
2.3 Expanded Reaction and Substrate Diversity 6

3 Supplementary Section 3 - Model-Guided Data Production **7**
3.1 Overview 7
3.2 Virtual BH Reaction Space 7
3.3 RBMAL acquisition function and batch construction 8
3.4 Iterative loop and retraining schedule 9

4 Supplementary Section 4 - BH HTE Literature Integration **11**
4.1 Compound–Reaction Diversity Score (CRDS) 11
4.2 Comparison with Simplified CRDS and Sample Size 11
4.3 Exponent Selection, Scope, and Limitations 12
4.4 Models and Training 14
4.5 Confidence Score Calculation 15

5 Supplementary Section 5 - Production Model Hyperparameters **19**
5.1 Motivation 19
5.2 Grid Search Protocol 19
5.3 Results 19
5.4 Model Selection Rationale 20
5.5 Production Model 21
5.6 Reagent Recommender Workflow 21

1 Supplementary Section 1 - Data Generation via HTE

1.1 Comparative Analysis of 96wp workflow and 384wp workflow

In the validation process, six substrate pairs and 64 reaction conditions were employed. The reactions within the 384 wp format were executed according to the layout depicted below, following the procedure outlined earlier. Subsequently, we compared these results with those obtained from the 96 wp, also following the previously described procedure. To facilitate easier comparison, the results from the 96 wp were reformatted to align with the 384-well layout. In conclusion, out of the 384 reactions, 228 exhibited differences of less than 5

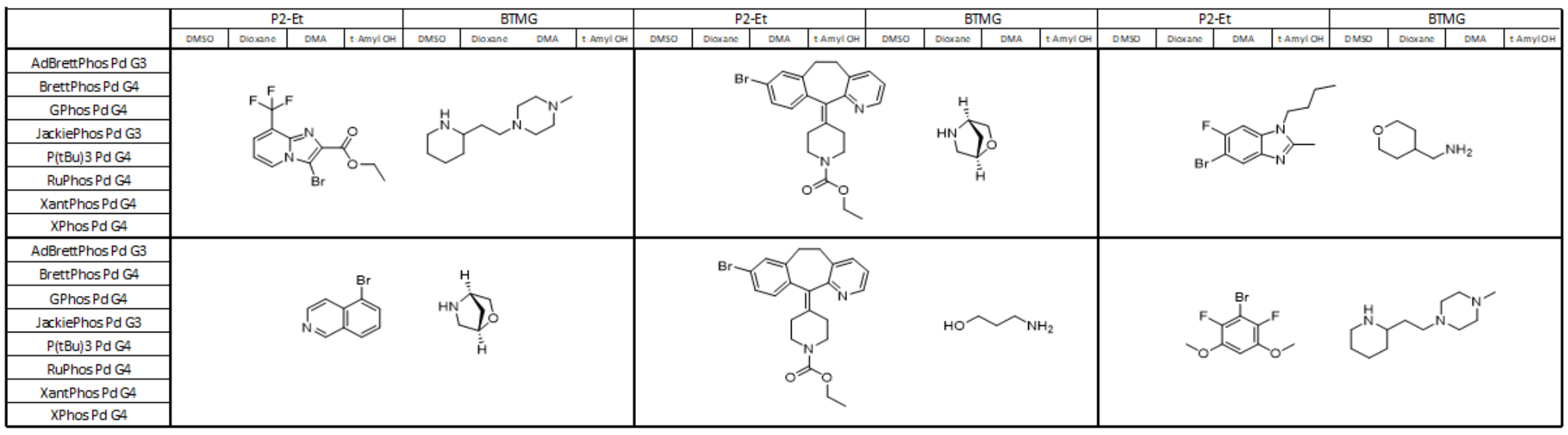

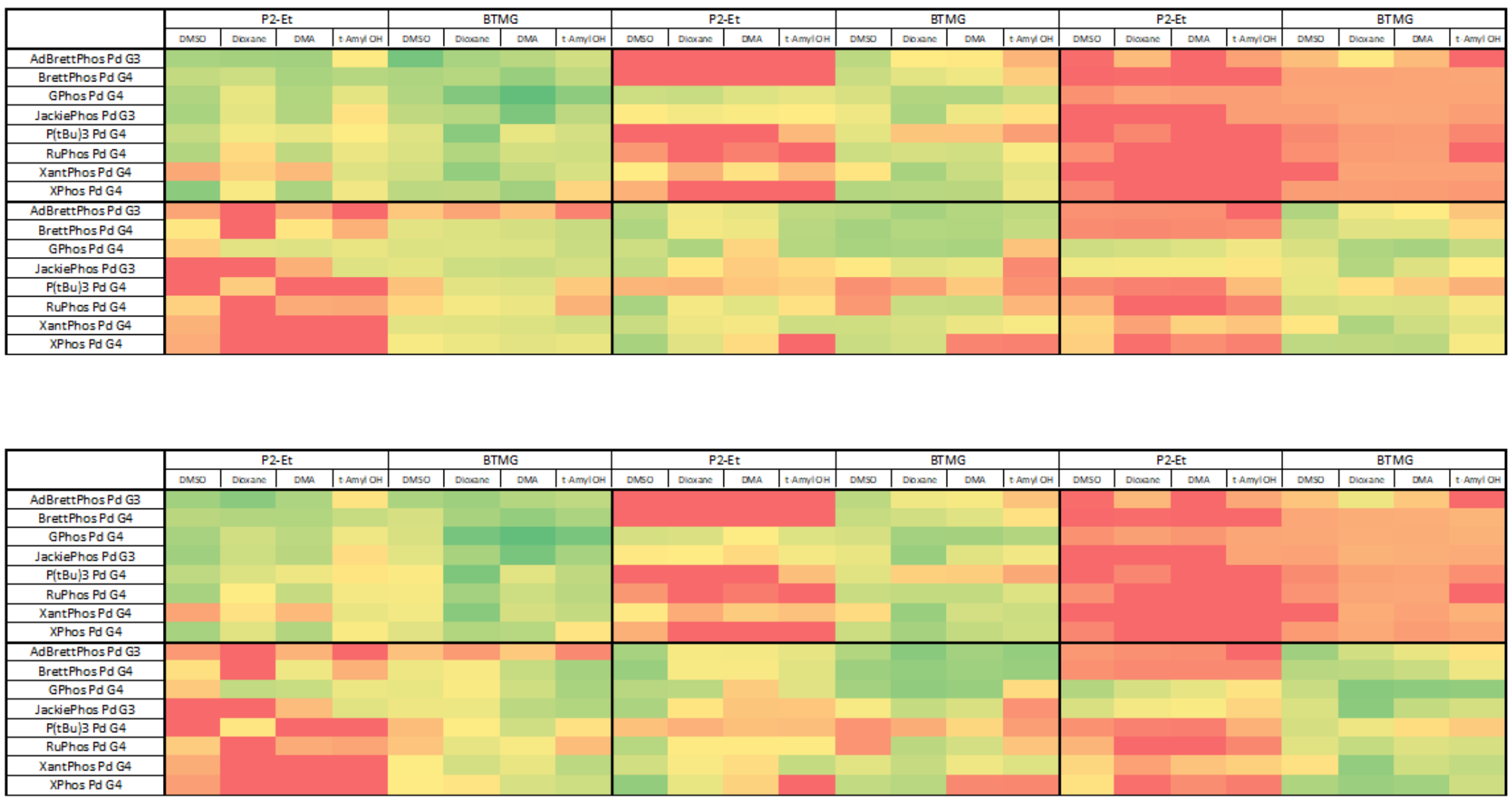

Supplementary Figure 1: Heatmap of the HTE result from 96 well plate and 384 well plate.

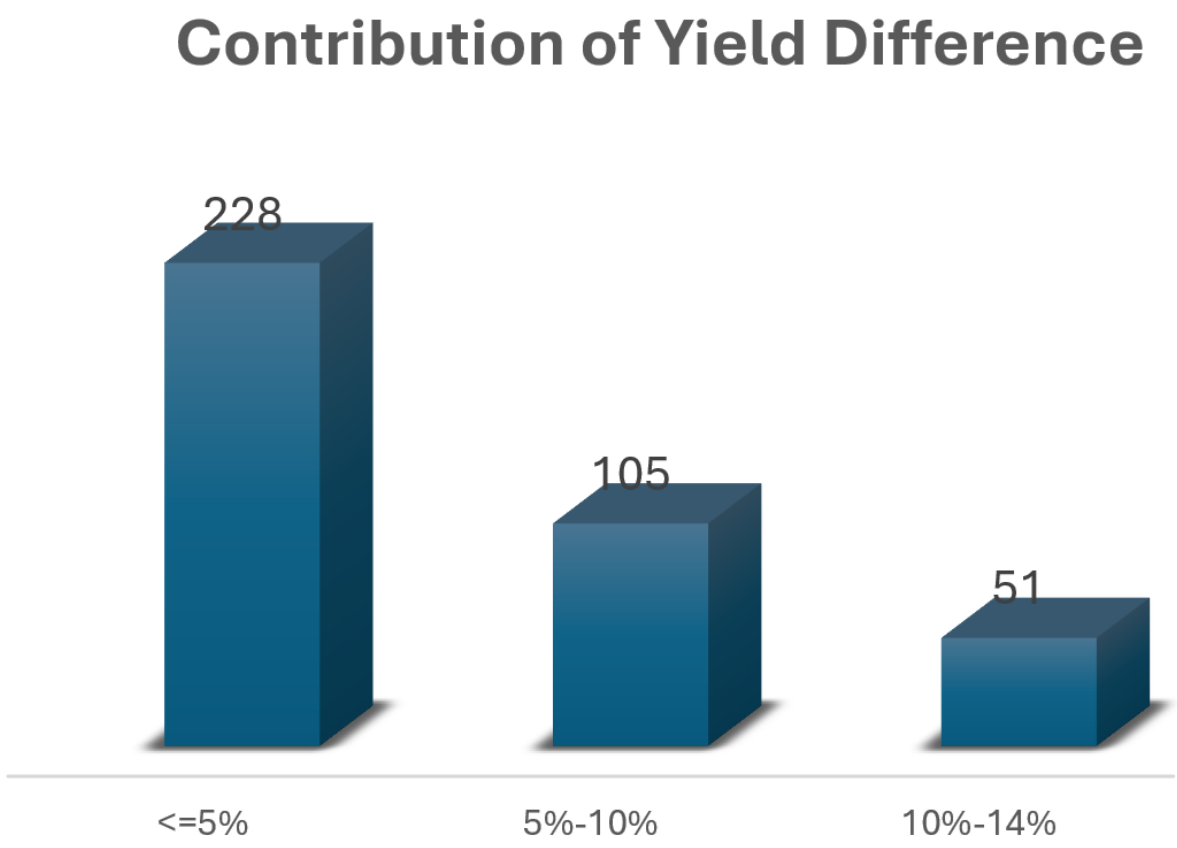

Supplementary Figure 2: Yield distribution in plate. Histogram showing the distribution of yield differences between 96-well and 384-well plate formats across validation reactions, demonstrating comparable quantification performance between the two experimental setups.

2 Supplementary Section 2 - Seed Data Selection and Production

The primary objective of this study was to develop a predictive model capable of accurately forecasting Buchwald-Hartwig (BH) reaction outcomes on out-of-distribution (OOD) data. Achieving reliable OOD prediction is essential for synthetic and medicinal chemistry, where diverse reagent combinations are regularly encountered. To meet this goal, we employed a two-stage approach: an initial data production phase to develop a baseline model, followed by a model-guided data production phase, where insights (uncertainty) from the model informed further experimentation to enhance predictive accuracy across broader substrate and reagent spaces.

2.1 Initial Data Production and Baseline Model

To build a foundational model capable of accurately predicting Buchwald-Hartwig (BH) reaction outcomes, we began by producing a high-quality, standardized dataset through high-throughput experimentation (HTE). Unlike data typically obtained from electronic lab notebooks (ELNs), which often lack consistency due to variations in reaction setup, data range, and purification methods, HTE enables standardized reaction conditions and analytical methods. This standardization ensured a high level of reproducibility across a broad set of reaction parameters, including ligand, pre-catalyst, solvent, base, and substrate, all of which substantially impact BH reaction yields. By selecting commercially available Buchwald palladacycle precatalysts, we reduced the reaction space to a manageable scale and allowed for consistency across experiments.

2.2 First Selection and Data Generation

In the first round of data production, we focused on selecting a diverse and representative set of substrates. Specifically focused on aryl bromides, given their ubiquity in our internal work and their relatively high reactivity in cross-coupling.

We started with an initial informer set selection of 76 hand-picked aryl halides, representing scaffold-like structures relevant to pharmaceutical applications. To further define the aryl halide space, we conducted a substructure pattern search, filtering out undesired structures and converting the selected structures to 4096-bit Morgan fingerprints (FPs) at radius 4. This conversion enabled us to quantify structural diversity and check the distribution of the informer set across a combined vendor and proprietary chemical space. After identifying regions lacking representation, we performed random sampling from these uncovered areas and ranked the selected aryl halides by cluster density to ensure adequate coverage across the substrate space.

For the selection of amines, we applied a similar substructure pattern search to define the amine space and removed undesired structures, eg, Sulfonic Acids, Sulfonates, and Sulfonamides, compounds containing both Bromine and Nitrogen or Iodine and Nitrogen which could present issues with reactivity or stability.

The resulting amines were converted into 4096-bit Morgan FPs at radius 3 to assess diversity. Sampling was performed across the entire amine space, and final selections were ranked by cluster density to prioritize amines representative of more densely populated clusters. This approach ensured that selected amines provided broad coverage of the chemical space, supporting the model's ability to generalize across diverse substrates.

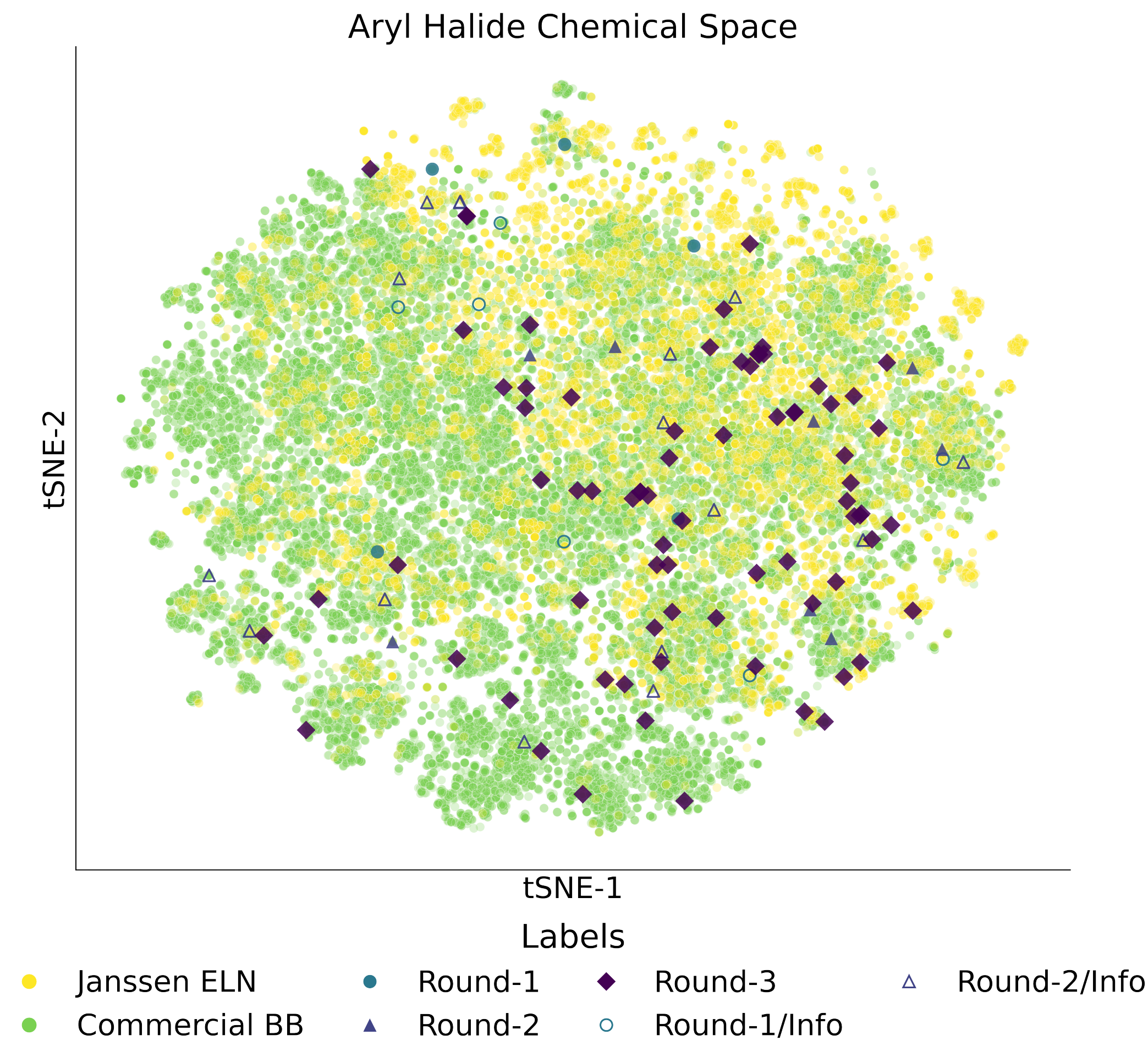

Supplementary Figure 3: tSNE plot of Aryl Halides selected across different rounds, in commercial and Janssen space.

The choice of different Morgan fingerprint radii for aryl halides (radius 4) and amines (radius 3) reflects the structural characteristics of each substrate class. Aryl halides on average possess more diverse and structurally extended aromatic scaffolds, and therefore benefit from a larger Morgan radius, which captures longer-range substituent and ring system context when assessing chemical diversity. In contrast, the amines included in this study are generally smaller, more modular structures, for which a radius of 3 is sufficient to encode meaningful structural variation without overspecifying the fingerprint. This choice avoids over-fragmentation of the amine chemical space while ensuring that aryl halide scaffolds are represented with an appropriately rich structural context. These radius settings were used exclusively for diversity analysis and clustering during seed selection.

Additionally, we chose 12 substrate pairs for which the substructures were underrepresented in our ELN data resulting in a set of 2,304 unique BH reactions, incorporating 8 catalysts, 6 bases, and 4 solvents. Product quantification was performed using charged aerosol detection (CAD) to accurately measure yields across various products. This initial round of data collection, although systematically diverse, resulted in low predictive power, indicating a need for a broader substrate scope.

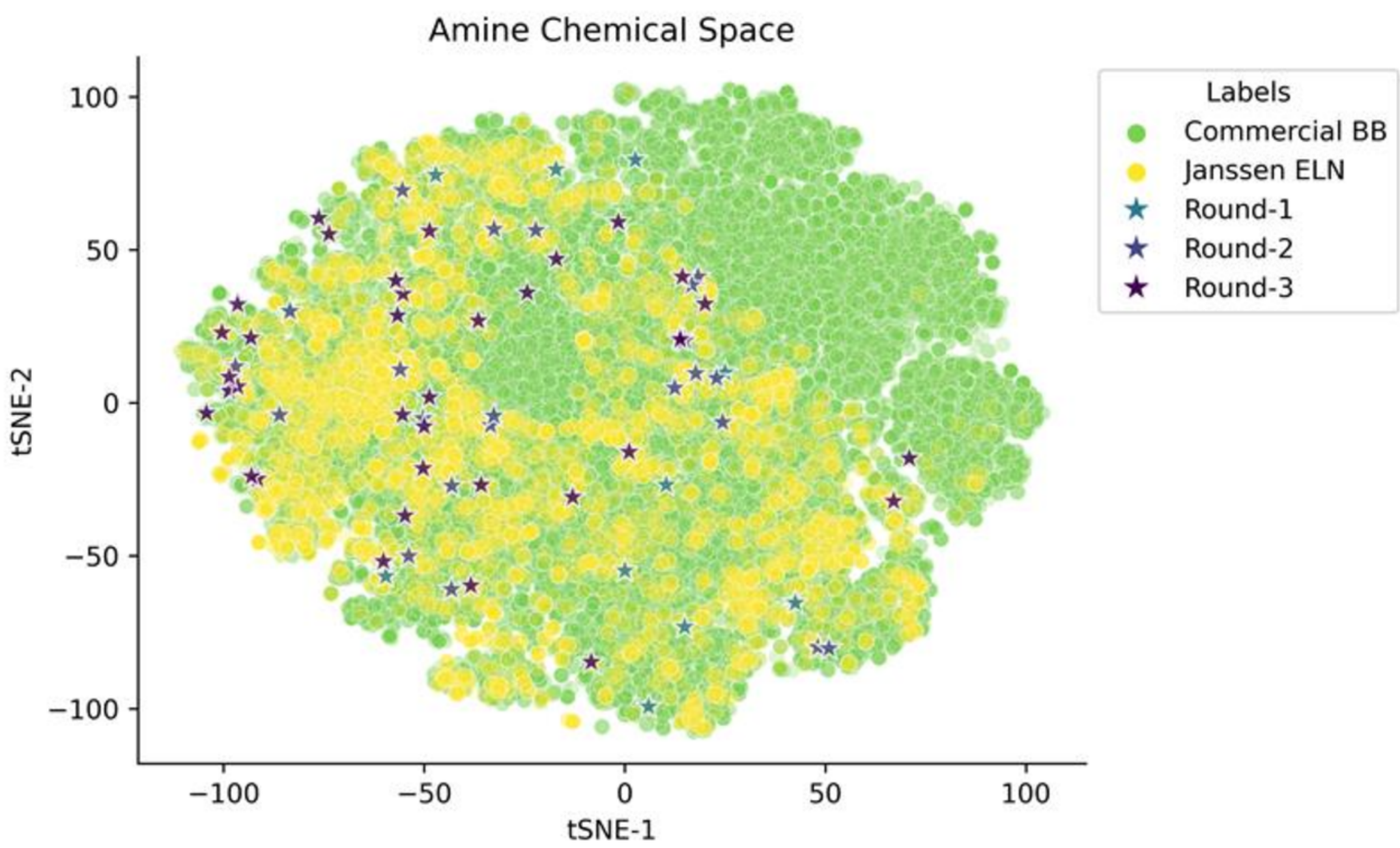

Supplementary Figure 4: tSNE plot of Amines selected across different rounds, in commercial and Janssen space.

2.3 Expanded Reaction and Substrate Diversity

To improve the performance and scope of the seed model, a second round of data collection expanded both the substrate diversity and reaction conditions. We selected a matrix of 24 aryl halides and 24 amines, incorporating 32 Buchwald palladacycle precatalysts, 6 bases, and 4 solvents, ultimately producing 3,840 reactions. Each pair of coupling partners was tested under multiple conditions (4 to 8 randomly selected combinations of catalysts, bases, and solvents) to capture a wider spectrum of possible outcomes.

The experimental workflow was optimized for automation, using polypropylene 384-well plates at a 1 µmol scale to facilitate high-throughput reaction setup and analysis. Automated liquid handling robots (SPT Dragonfly) were employed to streamline this process, enabling the completion of reactions within approximately 20 days. This expanded dataset substantially enhanced the model's predictive performance, and uncertainty estimation, providing greater diversity in reaction outcomes and substrate interactions, which proved essential for achieving reliable OOD predictions. The complete curated dataset, along with all code necessary to reproduce the results, is available at https://github.com/schwallergroup/bh-hte-ood and archived at https://zenodo.org/records/17634928.

The first stage of data production thus established a robust and diverse foundation for our BH model by systematically expanding the substrate and reaction conditions. Through carefully selected rounds of HTE-driven data collection, this stage laid the groundwork for a model capable of generalizing across unseen reagent combinations, setting the stage for subsequent refinement and model-guided data production in Stage 2.

3 Supplementary Section 3 - Model-Guided Data Production

3.1 Overview

Stage 2 operationalized active learning over a virtual Buchwald-Hartwig (BH) reaction space with 65.6M candidate reactions using a BERT-Enriched Embeddings (BEE) success estimation model fine-tuned on Stage 1 seed data. New experiments were selected in random batch mode active learning (RBMAL) with an acquisition function that (i) targets model uncertainty for specific, fully specified reactions (ArX, amine, Pd precatalyst/ligand, base, solvent), (ii) enforces diversity via Tanimoto distance from historical + in-batch selections, and (iii) respects substrate-space density and pharmaceutical applicability constraints. Substrate choices followed Stage 1 criteria (diversity + density + applicability); RBMAL prioritized reagents/conditions for those substrates.

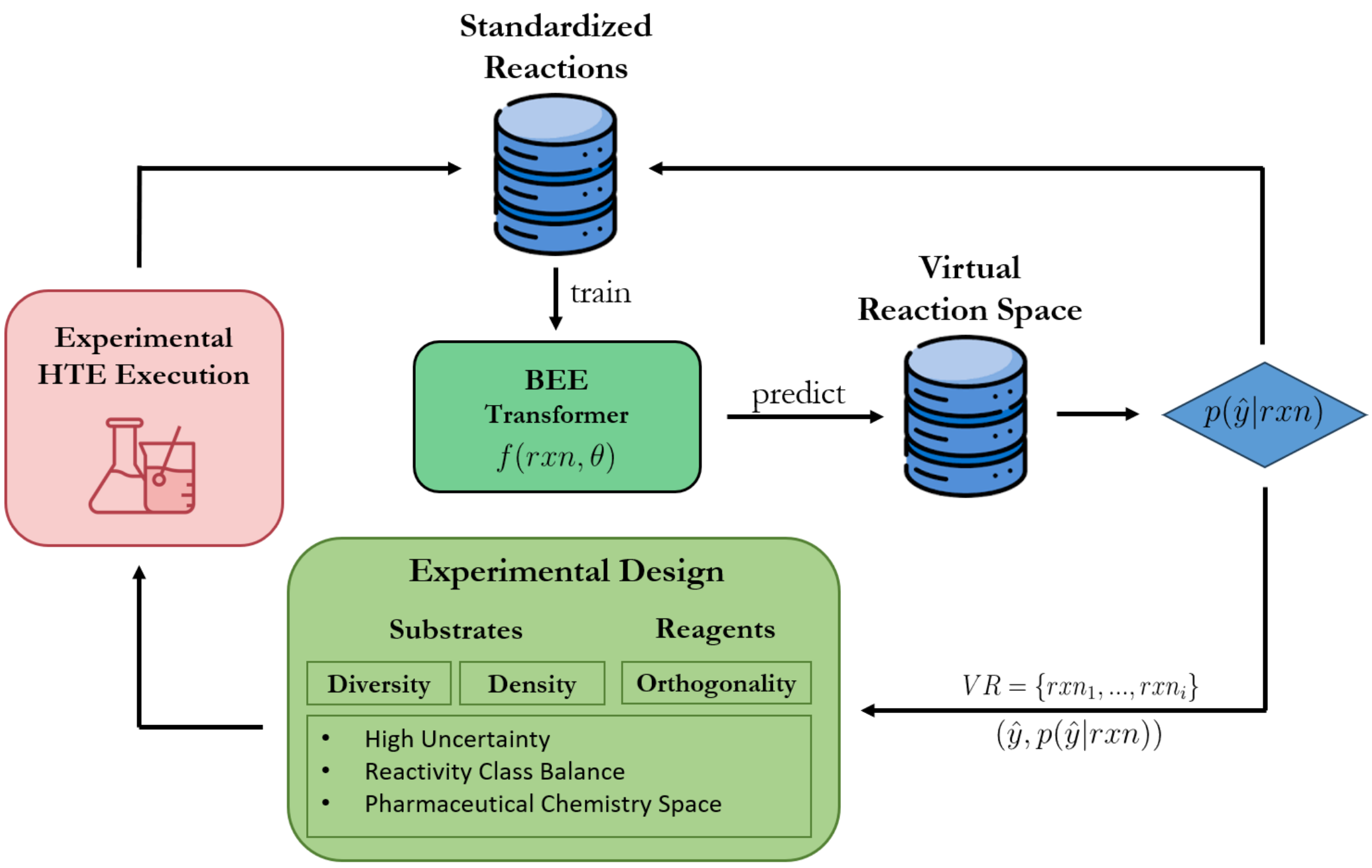

Supplementary Figure 5: Diagram with active learning flow. Schematic representation of the iterative active learning cycle showing data production, model training, uncertainty-based selection, and experimental validation steps used to strategically expand chemical space coverage.

3.2 Virtual BH Reaction Space

We enumerated a constrained, pharmaceutically relevant BH space by Cartesian product of curated dictionaries:

- Aryl halides (Ar-X): N = 186
- Amines (NuH): N = 57

- Pd sources/ligand systems: N = 43 Buchwald palladacycles: AdBrettPhos Pd G3, BrettPhos Pd G4, GPhos Pd G4, JackiePhos Pd G3, P(tBu)3 Pd G4, RuPhos Pd G4, XantPhos Pd G4, XPhos Pd G4, APhos Pd G4, cataCXium Pd G4, CPhos Pd G3, CyJohnPhos Pd G4, DavePhos Pd G3, DPPF Pd G4, DTBPF Pd G3, EPhos Pd G4, JosiPhos SL-J009-1 Pd G3, Me4tBuXPhos Pd G3, MorDalPhos Pd G4, N-XantPhos Pd G4, (o-tol)3 Pd G2, PCy3 Pd G4, QPhos Pd G3, rac-BINAP Pd G4, Pd G3, SPhos Pd G2, SPhos Pd G4, Pd G3, tBuDavePhos Pd G4, tBuXPhos Pd G3, TrixiePhos Pd G3, XPhos Pd G3, MeCgPPh Pd G3, Neopentyl(t-Bu)2P Pd G4, (t-Bu)2PhP Pd G4, Me3(OMe)tBuXPhos Pd G3, (t-Bu)PhCPhos Pd G4, VPhos Pd G4, (tBu)2PMe Pd G4, (R)-TolBINAP Pd G4, EtCPhos Pd G3, NJackiePhis Pd G4, (tBu)2P(nBu) Pd G4
- Bases: N = 12: P2-Et, Cs2CO3, NaOtBu, LiHMDS, NaBHT, BTMG, NaOTMS, DBU, KOPiv, K3PO4, MTBD, Cy2NMe
- Solvents: N = 12: t-Amyl OH, DMSO, DMA, 1,4-Dioxane, Toluene, MeCN, 2-Me-THF, DMF, CPME, NMP, iPrOAc, DME

Substrate selection followed Stage 1 logic:
cluster substrates (aryl ring system for halides; substructure for amines),
filter clusters absent from ELN (to ensure translational relevance),
rank candidates by cluster density (coverage proxy) and pharmaceutical applicability (availability/IP/safety),
pick cluster representatives with varying complexity.
After structural/availability/toxicity filters, enumeration yielded 10,602 potential products and 65.6M fully specified reactions (product, condition tuple). To account for diversity, we clustered the substrates represented with Morgan (ECFP) fingerprints (Ar-X: radius 4, 4096 bits; amines: radius 3, 4096 bits), as in Stage 1.
Once the substrate panel for an iteration was fixed, RBMAL optimized reagents/conditions.

3.3 RBMAL acquisition function and batch construction

Ranked batch-mode active learning (RBMAL) is an acquisition function that combines a model's uncertainty and a distance metric into a single score, as defined in Eq. 1. The score is calculated as:

$$\text{Score} = \alpha \cdot (1 - \text{Similarity}) + (1 - \alpha) \cdot \text{Uncertainty} \tag{1}$$

The parameter α is given by:

$$\alpha = \frac{|U|}{|U| + |D|} \tag{2}$$

where U and D represent the unlabeled and labeled sets, respectively.
In our study, we used the Tanimoto distance computed from Morgan fingerprints as the similarity measure. To select substrates, all 186 aryl halides were first ranked using Eq. 1, based on the mean confidence from 65 million predicted reactions. The top 75 aryl halides were then selected. For each aryl halide, we recursively selected 4 unique amines from a short-listed set of 57 amines, and 16 conditions for each pair, again utilizing Eq. 1 at both selection steps to ensure maximum diversity and informativeness.
The final experimental set included the 75 selected aryl halides, 37 amines, and a total of 714 combinations of catalyst, base, and solvent conditions.

3.4 Iterative loop and retraining schedule

Each active learning iteration followed this sequence:

- Freeze Transformer (BEE) trained on Stage 1 + all prior Stage 2 data.
- Score the virtual space restricted to the current substrate panel; build RBMAL batches as described in the subsection "RBMAL acquisition function and batch construction" above.
- Execute HTE (384-well or 96-well plates): automated setup, CAD/UPLC-MS readouts, standardized processing.
- Ingest data to tabular format: structure standardization, add role columns, canonicalize smiles and prepare into BEE compatible format.
- Retrain/fine-tune BEE with early stopping and validate on OOD substrates.

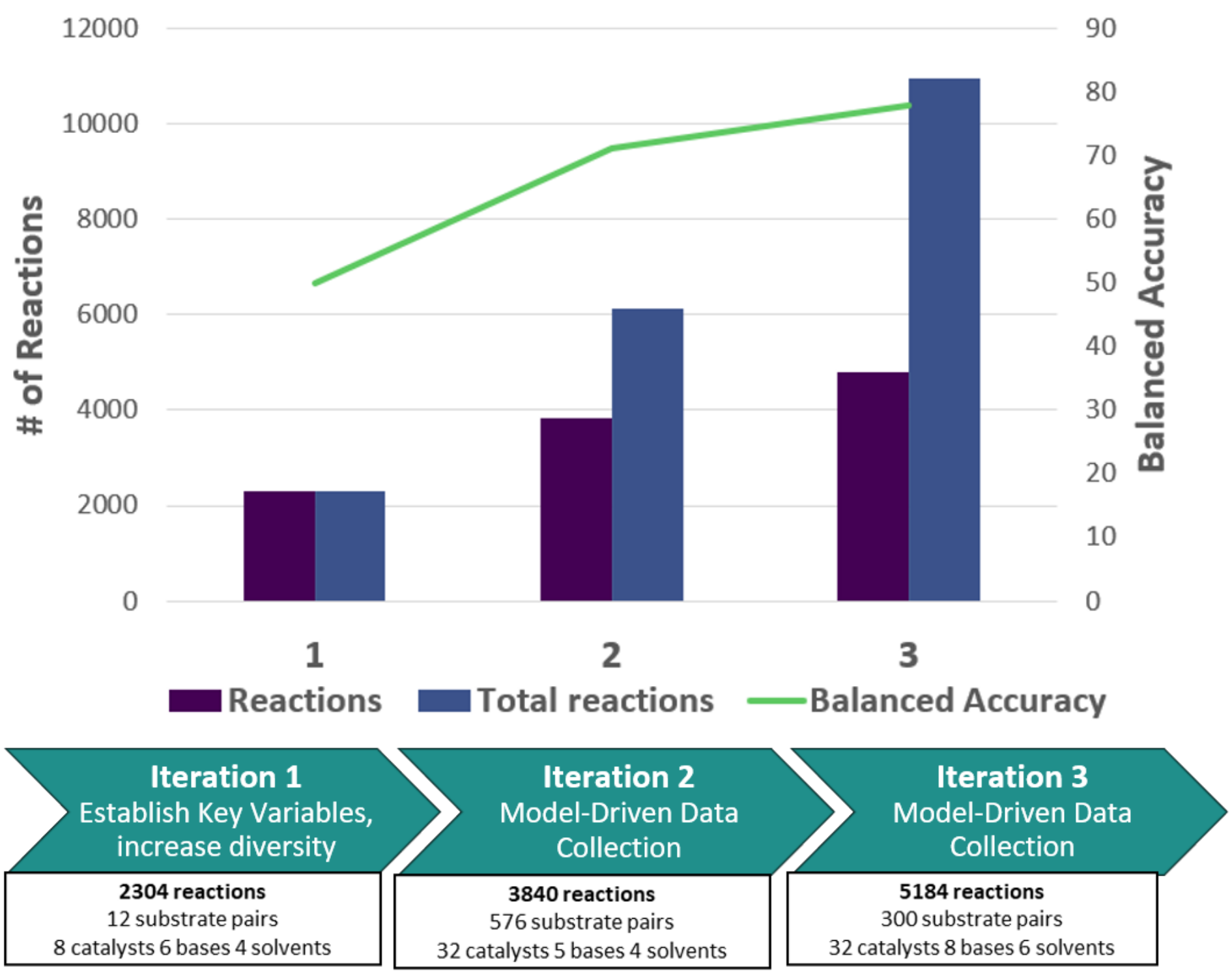

Supplementary Figure 6: Bar plot with number of reactions executed per iteration, and average balance accuracy across 20 train-test splits where substrates in test are not present in the train split.

In iteration2, the first model driven data collect, we substantially increased catalyst coverage and produced 3840 reaction samples. The resulting model was validated by creating 20 train-test splits where any substrate selected for the test were removed from the train, while the conditions are within domain for the model. The result of iteration 2 showed the first measure of generalizability for unseen substrates with a balanced accuracy of 72%. The following iteration we maintained the reagent scope with a small increase in base and solvents and produced our largest batch of 5184 reactions. The resulting

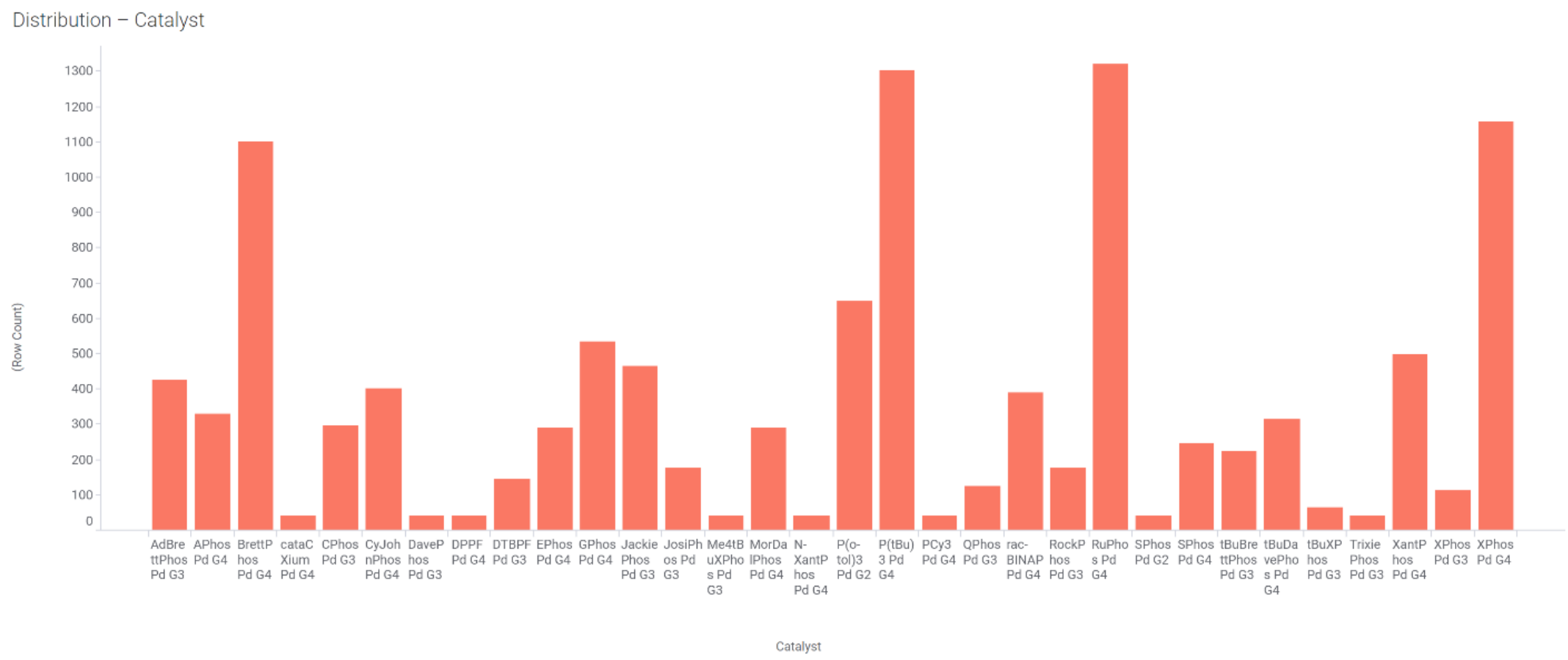

Supplementary Figure 7: Catalysts used and respective sample count. Bar chart displaying the distribution of reaction samples across 32 palladium precatalysts in the training dataset, illustrating the breadth of catalyst coverage and relative representation of each system.

model showed an improvement in performance when predicting unseen substrates with a balanced accuracy of 80%.

It's worth noting that despite having an ambitious reagent space coverage not all reagents were equally screened, with 9 of the 32 catalysts having a sample count under 100. Due to the nature of the OOD validation already described, these catalysts are represented according to the distribution in the train set and have low impact on performance metrics. Regarding the bases and solvents, all with exception of NaOTMS have a sample count above 500 and are well within domain of the covered reagent space.

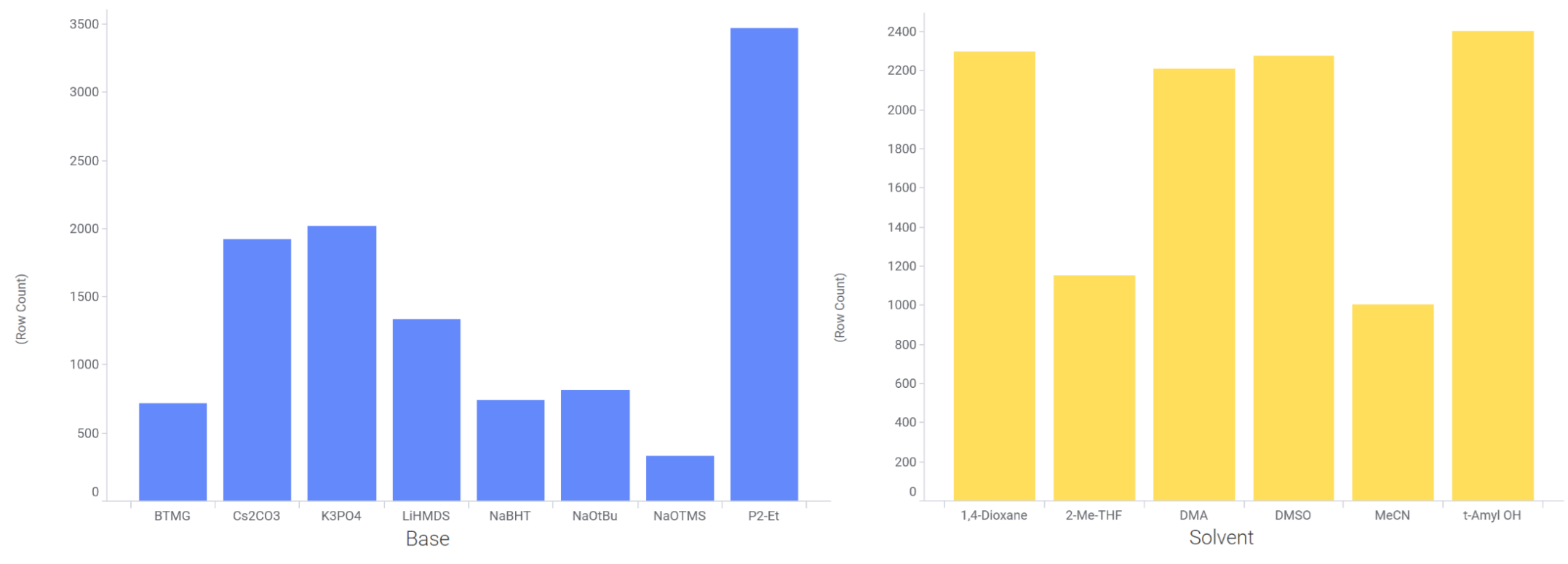

Supplementary Figure 8: Sample count of bases and solvents used.

4 Supplementary Section 4 - BH HTE Literature Integration

4.1 Compound–Reaction Diversity Score (CRDS)

To quantitatively assess the diversity of chemical datasets, we developed the **Compound–Reaction Diversity Score (CRDS)**. This metric integrates contributions from three key descriptors: (i) the number of distinct reactants (R_t), (ii) the number of distinct reagents (R_g), and (iii) the number of distinct reactions (R_n). Each descriptor is normalized against the maximum observed value within the collection and is weighted according to tunable exponents that allow dataset-specific prioritization.
The general form of CRDS is given by:

$$CRDS = \left(\frac{R_t}{R_t^{\max}}\right)^{\alpha} \times \left(\frac{\min(R_g, T_g)}{R_g^{\max}}\right)^{\beta_{\mathrm{u}}} \times \left(\max\left(\frac{R_g}{T_g}, 1\right)\right)^{\beta_{\mathrm{d}}} \times \left(\frac{\min(R_n, T_n)}{R_n^{\max}}\right)^{\gamma_{\mathrm{u}}} \times \left(\max\left(\frac{R_n}{T_n}, 1\right)\right)^{\gamma_{\mathrm{d}}}$$

Here,

- $R_t^{\max}, R_g^{\max}, R_n^{\max}$ denote the maximum values of reactants, reagents, and reactions across all datasets, ensuring normalization.
- $\alpha, \beta_{\mathrm{u}}, \beta_{\mathrm{d}}, \gamma_{\mathrm{u}}, \gamma_{\mathrm{d}}$ are weighting exponents that allow differential emphasis on each descriptor.
- T_g and T_n are *saturation thresholds*, beyond which additional diversity yields diminishing returns.

The structure of CRDS ensures that both breadth (coverage of reactants, reagents, and reactions up to the threshold values) and depth (expansion beyond the thresholds) are explicitly represented:

- For R_g and R_n, the "up" term ($\min(\cdot)$) emphasizes dataset growth up to the saturation threshold (T_g, T_n), rewarding balanced coverage.
- The "down" term ($\max(\cdot)$) introduces a diminishing-returns regime for values exceeding the threshold, preventing overinflation of the score by datasets dominated by excessive reagents or reactions without balanced representation elsewhere.

This design provides a nonlinear scoring scheme that prioritizes diverse yet balanced datasets, reflecting both practical chemical intuition (favoring expansion up to an optimal level) and statistical robustness (avoiding domination by single descriptors). The weighting exponents may be tuned to emphasize different chemical contexts, for example, increasing β_{up} to prioritize reagent diversity in synthetic methodology datasets, or increasing γ_{up} to emphasize reaction coverage in retrosynthetic benchmarks.

4.2 Comparison with Simplified CRDS and Sample Size

To test whether CRDS provides additional explanatory power compared to dataset size alone, we defined a **simplified CRDS** as:

$$CRDS_{\mathrm{simple}} = \left(\frac{R_t}{R_t^{\max}}\right)^{\alpha} \times \left(\frac{R_g}{R_g^{\max}}\right)^{\beta} \times \left(\frac{R_n}{R_n^{\max}}\right)^{\gamma}$$

This form omits the saturation thresholds and diminishing-returns terms, reducing the score to a straightforward normalized product of reactant, reagent, and reaction counts. To quantify the relationship between dataset characteristics and out-of-domain model performance, we computed Pearson and Spearman correlation coefficients between predictive accuracy and (i) dataset sample size, (ii) simplified CRDS, and (iii) full CRDS. The results are summarized in Supplementary Table 1.

Supplementary Table 1: Performance Metrics - Pearson and Spearman Correlations. Correlation coefficients between dataset characteristics (CRDS, simplified CRDS, and dataset size) and out-of-distribution model performance metrics (ROC AUC, balanced accuracy, F1 score, AU-PR-C), demonstrating the superiority of CRDS over dataset size alone for predicting OOD performance.

	**Pearson**				**Spearman**			
	**ROC AUC**	**Bal. Acc.**	**F1 Score**	**AU-PR-C**	**ROC AUC**	**Bal. Acc.**	**F1 Score**	**AU-PR-C**
CRDS	**0.790**	**0.770**	**0.308**	0.170	0.452	**0.429**	0.310	0.286
Simplified CRDS	0.534	0.558	0.263	**0.381**	0.262	0.214	**0.429**	**0.405**
Dataset Size	0.603	0.541	0.287	0.062	**0.476**	0.405	-0.214	-0.310

These results demonstrate that while dataset size correlates weakly with out-of-domain performance, incorporating diversity-aware descriptors via CRDS produces a substantially stronger association. This underscores the value of CRDS as a principled metric for benchmarking dataset utility in chemical machine learning.

For reference, Supplementary Table 2 lists the CRDS values alongside dataset composition and best OOD performance for each training corpus. Note that CRDS is a relative score: because the normalization denominators are derived from the full collection of datasets under comparison, the absolute values would change if different datasets were included.

Supplementary Table 2: Dataset composition and CRDS for each training corpus.

**Training Corpus**	**Substrate Pairs**	**Reagent Combos**	**Samples**	**CRDS**
Chem.S.23	469	147	750	0.006
Sci.15	8	96	768	<0.001
Chem.S.16	18	7	144	<0.001
Sci.18	15	12	4312	<0.001
Nat.C.24	27	779	2632	0.002
Sci.23	123	176	3359	0.007
JACS.25	4204	1	4204	0.100
JnJ25	888	1137	11328	0.119
All w/o JnJ25	4864	1218	12037	0.209
All w JnJ25	6011	2351	23693	0.337

4.3 Exponent Selection, Scope, and Limitations

The weighting exponents (α, β_u, β_d, γ_u, γ_d) and saturation thresholds (T_g, T_n) were fitted empirically by maximizing the Pearson correlation between CRDS and observed OOD model performance (ROC AUC) across the eight Buchwald-Hartwig HTE data sources used in this study. The values used are: $\alpha = 1$, $\beta_u = 0.2$, $\beta_d = 0.1$, $\gamma_u = 0.9$, $\gamma_d = 0.2$,

$T_g = 4000$, and $T_n = 5000$. The rationale is that a useful diversity score should track downstream predictive utility: datasets that are more diverse in a chemically meaningful way should produce models that generalize better, assuming sample size is sufficient for model fitting.
Because CRDS is a multiplicative score across reactant, reagent, and reaction contributions, it intentionally favors balanced diversity over sheer volume. This is a deliberate design choice motivated by the observation that dataset size alone is a poor predictor of OOD performance (Pearson $r = 0.60$ for dataset size vs. $r = 0.79$ for CRDS against ROC AUC; Supplementary Table 1).
An important caveat is that the exponents encode assumptions about what constitutes sufficient reagent and reaction coverage for this specific reaction family. Applying CRDS to a different reaction type (for example, Suzuki coupling) would require reparameterization of the exponents against OOD performance data for that reaction, as the combinatorial space, role structure, and sensitivity to individual reagents differ fundamentally. Furthermore, the behavior of CRDS on datasets that mix multiple reaction types is untested. In such cases, the data requirements for OOD coverage scale differently across reaction types, and the meaningfulness of a single CRDS score would heavily depend on the transferability of learned representations between the reaction types present in the dataset. We therefore recommend CRDS as a parameterized framework for within-reaction-type dataset benchmarking, where the exponents should be recalibrated for each different reaction family.

S4.2 Literature HTE Sources for OOD Performance

There were two goals to be addressed in the in-silico validation portion of this project:

- To quantify how informative each individual open-source BH HTE dataset is for training models that generalize to unseen substrates.
- To benchmark All w/o JnJ25 (all literature sources combined, excluding our open source HTE data) and All w JnJ25 (all literature + HTE data produced in this project).

Towards that end we trained multiple ML models per training corpus and evaluated them on outer clusters that are sparsely represented by the training corpus, over multiple random cluster initializations and calculate Mean $\pm$ std per metric. To prepare the data we operated on a pre-curated table in which chemical entities were normalized across sources (beyond canonicalization), ensuring that the same reagent has a single, unified representation in the final dataset. This greatly impacts ability for models to generalize across external data sources. Each row encodes the source of the sample (Source column) and a single reaction with its unified entity columns (Aryl Halide, Amine, Pd system, Base, Solvent) which inherently labels role information. Role is an interesting variable which allows different encoding strategies of the chemical entity depending on role, an opportunity for model improvement in future work.
It's worth noting that this cross-source normalization step is only relevant when combining the external sources, during the acquisition stage of the active learning cycle we did not rely on this harmonization.

- **Reactivity class**: The models trained were exclusively binary classification models, and we defined the positive class as reactions that yielded over 10% producing $labels \in \{0, 1\}$.

- **Features**: Due to the large number of training sets we wanted to test (10), and the combinatorial increase with 10 random cluster initializations we opted for Reaction Fingerprints, DRFP with $radius = 3$ and $nBits = 2048$ and Physicochemical features (eg, TPSA, HOMO LUMO Gap, LogP, MolarRefractivity, MinPartialCharge, MaxPartialCharge, NumRotatableBonds). For this particular benchmark it was not practical to train-test large models like BEE hundreds or thousands of times ($10 \times 10 \times 6\,\text{models} \times N\,\text{experiments}$).

- **Clustering and OOD evaluation protocol**: We used k-means clustering in reaction/feature space (DRFP, including reagents) to create structure-aware outer-cluster tests, with K = 12 clusters. For each training corpus S (a single literature source) we count the number of training examples per cluster and define outer clusters as those where S contributes 30 examples. We also remove any samples in the cluster tests containing a substrate already present in the training data. This train-test split is indicated in the tables bellow as DRFP OOD Clusters - S.R., a highly challenging OOD regime since reagents are considered in cluster formation and no substrate leakage is allowed. To compare the performance difference between combining JnJ25 to all existing data, we used DRFP OOD Clusters, the methodology is the same as the one for individual sources, but to ensure all available chemical space is covered in the test samples are not removed. This creates an approximate OOD regime: predictions are scored on clusters that are poorly covered by the training corpus, reducing in-domain leakage.

4.4 Models and Training

For each corpus S and run, we train six classifiers on DRFP features using a common training routine (scalers, where required, are fit only on the training data):

- Random Forest (`rf_model`)
- Gradient Boosting (`gradient_boosting_model`)
- Gaussian Naive Bayes (`GaussianNB`)
- Logistic Regression (`lr_model`)
- Multi-Layer Perceptron (`mlp_model`)
- k-Nearest Neighbors (`knn_model`)

Training corpora S: (i) each literature `Source` individually; (ii) **(All w/o JnJ25)**: union of all literature sources excluding our HTE data; (iii) **All w JnJ25**: union of all literature sources plus our HTE data. The same clustering protocol applies in all cases in (i), and outer clusters are defined relative to S. For (ii) and (iii) we select 9 clusters for the train set and 3 clusters for the test set.

To evaluate the models and reduce the possible bias introduced by any given metric we calculated performance on the outer-cluster evaluation set, across multiple metrics:

- Balanced Accuracy (BA)
- ROC AUC

Model	Hyperparameter	Value
Random Forest	Number of estimators (`n_estimators`)	300
	Maximum depth (`max_depth`)	None
	Minimum samples split (`min_samples_split`)	4
	Minimum samples leaf (`min_samples_leaf`)	3
	Splitting criterion (`criterion`)	Entropy
Gradient Boosting	Number of estimators (`n_estimators`)	100
Gaussian Naive Bayes	Max Feature Variance Added (`var_smoothing`)	1e-09
Logistic Regression	Maximum iterations (`max_iter`)	400
Multi-Layer Perceptron (MLP)	Hidden layer sizes (`hidden_layer_sizes`)	(100, 20)
	Solver (`solver`)	L-BFGS
k-Nearest Neighbors	Number of neighbors (`n_neighbors`)	5

Supplementary Table 3: Hyperparameter settings for benchmark models. These near-default configurations were kept constant across all training corpora and feature sets to ensure fair, unbiased comparison of data sources. Hyperparameter optimization for the production model is described in Supplementary Section 5.

- F1 score (positive class = 1)
- F0 score (positive class = 0)
- AU-PR-C (area under precision–recall curve for class 1)
- Precision (class 1)
- Recall (class 1)

Binary thresholding affects class balance, therefore we report both threshold-insensitive metrics (ROC-AUC, AU-PR-C) and threshold-dependent ones (F1, BA). In order to tackle stochastic bias, we perform 10 independent runs (`run = 0..10`), each with a new k-means `random_state=run`. For each (S, model) we train on all rows from S (shuffled with `random_state_key=run`), evaluate on that run's outer clusters, and append metrics. The summary Supplementary Table 4 contains the average and standard deviation for each metric, for the best performing model in each train source and reaction features. For the full results table view Supplementary Table 5 and Supplementary Table 6.

4.5 Confidence Score Calculation

Model uncertainty was quantified through a confidence score derived from predicted class probabilities. For each reaction, the trained classifier outputs a probability $p \in [0, 1]$ representing the likelihood of reaction success (yield > 10%). This probability was transformed into a confidence score using:

$$\text{confidence} = |p - 0.5| + 0.5 \tag{3}$$

This transformation maps predicted probabilities to a confidence range of $[0.5, 1.0]$, where:

- confidence = 0.5 indicates maximum uncertainty ($p = 0.5$, equiprobable outcomes)
- confidence = 1.0 indicates maximum certainty ($p = 0.0$ or $p = 1.0$)

The absolute value operation ensures that both highly confident success predictions ($p \to 1$) and highly confident failure predictions ($p \to 0$) receive high confidence scores. This metric was used to rank reagent combinations for the experimental validations (Figure 5) and to partition predictions into five equally-spaced confidence bins ($[0.5, 0.6)$, $[0.6, 0.7)$, $[0.7, 0.8)$, $[0.8, 0.9)$, $[0.9, 1.0]$) for calibration analysis (Figure 4).
For example:

- A prediction of $p = 0.95$ (95% success probability) yields confidence $= |0.95 - 0.5| + 0.5 = 0.95$
- A prediction of $p = 0.05$ (5% success probability, 95% failure) yields confidence $= |0.05 - 0.5| + 0.5 = 0.95$
- A prediction of $p = 0.50$ (maximum uncertainty) yields confidence $= |0.5 - 0.5| + 0.5 = 0.50$

This confidence transformation enables prioritization of predictions where the model expresses high certainty in either outcome, which is particularly valuable for reagent recommendation and active learning workflows where experimental resources must be allocated efficiently.

Figure 3 — Dataset Composition and CRDS vs OOD Performance

Figure 3a — Dataset composition statistics
Source data file: `Fig_3a.csv`
Contents: Raw dataset composition metrics for each training corpus used in CRDS calculation and OOD benchmarking. Columns include unique reactant pairs, unique reagent combinations, total reaction samples, and dataset name.

Figure 3b,c,d — CRDS values and correlation analysis
Source data files:

- `table2_dataset_composition_crds.csv` — Dataset composition and computed CRDS values for all training corpora
- `table3_hyperparameters_benchmark.csv` — Hyperparameter settings for benchmark models used in OOD performance evaluation

The CRDS correlation values shown in Figure 3 panels are computed from the best OOD performance metrics (Supplementary Table 4) combined with CRDS values from `table2_dataset_composition_crds.csv`.

Figure 5 — Experimental Validation Results

Figure 5a — Rescue of previously unsuccessful substrate pairs
Source data file: `BH_Manu_NewConditions_20250310_result.csv`
Location: `data/` directory in repository
Contents: Experimental validation results (118 reactions) for model-guided rescue of substrate pairs that previously failed under tested conditions. Columns include substrate SMILES, predicted reagent combinations (catalyst, base, solvent), model confidence scores, prior failure count, and measured yields.

Figure 5b — Prospective validation on unseen substrates
Source data file: `Case1_selections_20250408_result.csv`
Location: `data/` directory in repository
Contents: Prospective experimental validation results (300 reactions) for model predictions on unseen aryl halide–amine pairs not present in training data. Columns include substrate SMILES, predicted reagent combinations, model confidence scores, clustering assignments, and measured yields.

Supplementary Table 4: Best Performance per Data Source. Summary of the top-performing model for each individual training corpus evaluated under strict out-of-distribution conditions (DRFP OOD Clusters with substrate removal), showing mean and standard deviation across 10 random cluster initializations for key classification metrics.

Model	Train Data	Features	Test Data	**ROC AUC**		**Balanced Accuracy**		**F1 Score**		**F0 Score**		**AU-PR-C**	
Model	Train Data	Features	Test Data	Avg	Std	Avg	Std	Avg	Std	Avg	Std	Avg	Std
gaussianNB_model	Chem.S.23	DRFP	DRFP OOD Clusters - S.R.	0.5	0.0	0.5	0.0	0.62	0.01	0.02	0.01	0.72	0.0
gaussianNB_model	Sci.15	PhysChem	DRFP OOD Clusters - S.R.	0.48	0.02	0.47	0.01	0.45	0.02	0.48	0.04	0.54	0.01
gaussianNB_model	Chem.S.16	PhysChem	DRFP OOD Clusters - S.R.	0.6	0.01	0.58	0.01	0.59	0.02	0.56	0.01	0.66	0.02
rf_model	Sci.18	PhysChem	DRFP OOD Clusters - S.R.	0.54	0.0	0.5	0.0	0.56	0.0	0.0	0.0	0.43	0.01
gaussianNB_model	Nat.C.24	DRFP	DRFP OOD Clusters - S.R.	0.53	0.0	0.53	0.0	0.6	0.01	0.41	0.01	0.69	0.01
knn_model	JnJ25	DRFP	DRFP OOD Clusters - S.R.	0.65	0.01	0.62	0.01	0.64	0.02	0.6	0.02	0.72	0.02
gaussianNB_model	JACS.25	PhysChem	DRFP OOD Clusters - S.R.	0.63	0.0	0.59	0.0	0.59	0.0	0.57	0.0	0.55	0.0
knn_model	Sci.23	DRFP	DRFP OOD Clusters - S.R.	0.51	0.0	0.5	0.0	0.57	0.01	0.3	0.0	0.58	0.01
knn_model	All w JnJ25	DRFP	DRFP OOD Clusters	0.81	0.01	0.75	0.01	0.71	0.01	0.78	0.01	0.79	0.02
knn_model	All w/o JnJ25	DRFP	DRFP OOD Clusters	0.65	0.03	0.62	0.02	0.6	0.03	0.61	0.03	0.63	0.03

Supplementary Table 5: All Performances of Data Sources Combined. Complete performance results for all six benchmark models trained on combined datasets (all sources with and without JnJ25 data) using DRFP and physicochemical features, evaluated on DRFP OOD clusters to assess the impact of including industrial HTE data.

Model	Features	Test Data	Train Data	**ROC AUC**		**Balanced Accuracy**		**F1 Score**		**F0 Score**		**AU-PR-C**	
Model	Features	Test Data	Train Data	Avg	Std	Avg	Std	Avg	Std	Avg	Std	Avg	Std
rf_model	DRFP	DRFP OOD Clusters	All with JnJ	0.8	0.02	0.71	0.02	0.59	0.06	0.8	0.01	0.77	0.02
gradient_boosting_model	DRFP	DRFP OOD Clusters	All with JnJ	0.78	0.01	0.7	0.02	0.61	0.04	0.78	0.01	0.75	0.02
lr_model	DRFP	DRFP OOD Clusters	All with JnJ	0.67	0.02	0.62	0.02	0.56	0.03	0.67	0.03	0.62	0.02
mlp_model	DRFP	DRFP OOD Clusters	All with JnJ	0.74	0.03	0.68	0.03	0.6	0.05	0.74	0.02	0.7	0.02
knn_model	DRFP	DRFP OOD Clusters	All with JnJ	0.81	0.01	0.75	0.01	0.71	0.01	0.78	0.01	0.79	0.02
gaussianNB_model	DRFP	DRFP OOD Clusters	All with JnJ	0.7	0.04	0.63	0.04	0.5	0.1	0.73	0.02	0.68	0.04
rf_model	PhysChem	DRFP OOD Clusters	All with JnJ	0.4	0.02	0.5	0.0	0.02	0.03	0.71	0.01	0.4	0.01
gradient_boosting_model	PhysChem	DRFP OOD Clusters	All with JnJ	0.77	0.01	0.69	0.02	0.6	0.03	0.76	0.02	0.73	0.02
lr_model	PhysChem	DRFP OOD Clusters	All with JnJ	0.67	0.02	0.63	0.02	0.54	0.04	0.69	0.02	0.61	0.02
mlp_model	PhysChem	DRFP OOD Clusters	All with JnJ	0.75	0.02	0.69	0.01	0.63	0.02	0.74	0.02	0.7	0.02
knn_model	PhysChem	DRFP OOD Clusters	All with JnJ	0.75	0.02	0.69	0.02	0.63	0.03	0.75	0.02	0.72	0.03
gaussianNB_model	PhysChem	DRFP OOD Clusters	All with JnJ	0.65	0.02	0.62	0.01	0.55	0.02	0.67	0.02	0.58	0.02
rf_model	DRFP	DRFP OOD Clusters	All without JnJ	0.67	0.04	0.61	0.04	0.38	0.11	0.75	0.02	0.64	0.05
gradient_boosting_model	DRFP	DRFP OOD Clusters	All without JnJ	0.66	0.03	0.61	0.03	0.45	0.08	0.72	0.02	0.63	0.04
lr_model	DRFP	DRFP OOD Clusters	All without JnJ	0.51	0.02	0.52	0.02	0.42	0.06	0.57	0.03	0.45	0.02
mlp_model	DRFP	DRFP OOD Clusters	All without JnJ	0.57	0.04	0.56	0.02	0.38	0.07	0.65	0.03	0.54	0.04
knn_model	DRFP	DRFP OOD Clusters	All without JnJ	0.65	0.03	0.62	0.02	0.6	0.03	0.61	0.03	0.63	0.03
gaussianNB_model	DRFP	DRFP OOD Clusters	All without JnJ	0.54	0.02	0.53	0.02	0.28	0.09	0.63	0.03	0.57	0.04
rf_model	PhysChem	DRFP OOD Clusters	All without JnJ	0.44	0.03	0.5	0.0	0.01	0.02	0.73	0.02	0.39	0.02
gradient_boosting_model	PhysChem	DRFP OOD Clusters	All without JnJ	0.49	0.03	0.51	0.02	0.3	0.07	0.66	0.02	0.42	0.03
lr_model	PhysChem	DRFP OOD Clusters	All without JnJ	0.5	0.02	0.49	0.02	0.31	0.06	0.62	0.04	0.41	0.02
mlp_model	PhysChem	DRFP OOD Clusters	All without JnJ	0.48	0.03	0.49	0.02	0.43	0.05	0.5	0.05	0.41	0.03
knn_model	PhysChem	DRFP OOD Clusters	All without JnJ	0.47	0.02	0.49	0.01	0.36	0.04	0.6	0.02	0.43	0.03
gaussianNB_model	PhysChem	DRFP OOD Clusters	All without JnJ	0.58	0.05	0.56	0.03	0.37	0.08	0.68	0.03	0.5	0.05

Supplementary Table 6: All Performances per Data Source. Comprehensive performance results for all six benchmark models trained on each individual literature dataset using DRFP and physicochemical features, evaluated under strict OOD conditions with substrate removal, providing detailed comparison across data sources and feature types.

Model	Features	Test Data	Train Data	**ROC AUC**		**Balanced Accuracy**		**F1 Score**		**F0 Score**		**AU-PR-C**	
Model	Features	Test Data	Train Data	Avg	Std	Avg	Std	Avg	Std	Avg	Std	Avg	Std
rf_model	PhysChem	DRFP OOD Clusters - S.R.	Sci.15	0.48	0.02	0.5	0.0	0.0	0.0	0.74	0.02	0.48	0.07
gradient_boosting_model	PhysChem	DRFP OOD Clusters - S.R.	Sci.15	0.52	0.01	0.5	0.0	0.0	0.0	0.74	0.02	0.42	0.02
lr_model	PhysChem	DRFP OOD Clusters - S.R.	Sci.15	0.44	0.01	0.41	0.01	0.46	0.03	0.23	0.01	0.37	0.03
mlp_model	PhysChem	DRFP OOD Clusters - S.R.	Sci.15	0.47	0.01	0.48	0.01	0.46	0.02	0.47	0.02	0.55	0.02
knn_model	PhysChem	DRFP OOD Clusters - S.R.	Sci.15	0.53	0.01	0.51	0.0	0.16	0.01	0.72	0.02	0.44	0.01
gaussianNB_model	PhysChem	DRFP OOD Clusters - S.R.	Sci.15	0.48	0.02	0.47	0.01	0.45	0.02	0.48	0.04	0.54	0.01
rf_model	PhysChem	DRFP OOD Clusters - S.R.	Chem.S.16	0.56	0.03	0.51	0.02	0.04	0.15	0.7	0.03	0.55	0.1
gradient_boosting_model	PhysChem	DRFP OOD Clusters - S.R.	Chem.S.16	0.62	0.01	0.51	0.02	0.16	0.06	0.69	0.02	0.55	0.01
lr_model	PhysChem	DRFP OOD Clusters - S.R.	Chem.S.16	0.62	0.01	0.57	0.01	0.54	0.01	0.61	0.01	0.56	0.02
mlp_model	PhysChem	DRFP OOD Clusters - S.R.	Chem.S.16	0.62	0.02	0.59	0.01	0.56	0.02	0.63	0.01	0.65	0.02
knn_model	PhysChem	DRFP OOD Clusters - S.R.	Chem.S.16	0.55	0.01	0.55	0.01	0.36	0.01	0.68	0.02	0.53	0.02
gaussianNB_model	PhysChem	DRFP OOD Clusters - S.R.	Chem.S.16	0.6	0.01	0.58	0.01	0.59	0.02	0.56	0.01	0.66	0.02
rf_model	PhysChem	DRFP OOD Clusters - S.R.	Sci.18	0.54	0.0	0.5	0.0	0.56	0.0	0.0	0.0	0.43	0.01
gradient_boosting_model	PhysChem	DRFP OOD Clusters - S.R.	Sci.18	0.46	0.01	0.47	0.01	0.5	0.01	0.23	0.06	0.37	0.0
lr_model	PhysChem	DRFP OOD Clusters - S.R.	Sci.18	0.41	0.0	0.5	0.0	0.55	0.0	0.08	0.0	0.5	0.0
mlp_model	PhysChem	DRFP OOD Clusters - S.R.	Sci.18	0.39	0.0	0.45	0.0	0.5	0.0	0.12	0.01	0.51	0.01
knn_model	PhysChem	DRFP OOD Clusters - S.R.	Sci.18	0.48	0.01	0.49	0.01	0.52	0.01	0.25	0.04	0.52	0.06
gaussianNB_model	PhysChem	DRFP OOD Clusters - S.R.	Sci.18	0.49	0.0	0.55	0.0	0.34	0.0	0.73	0.0	0.44	0.0
rf_model	PhysChem	DRFP OOD Clusters - S.R.	Nat.C.24	0.55	0.02	0.54	0.02	0.63	0.04	0.37	0.12	0.55	0.04
gradient_boosting_model	PhysChem	DRFP OOD Clusters - S.R.	Nat.C.24	0.5	0.01	0.49	0.01	0.37	0.03	0.57	0.01	0.49	0.02
lr_model	PhysChem	DRFP OOD Clusters - S.R.	Nat.C.24	0.48	0.01	0.48	0.0	0.52	0.01	0.45	0.02	0.48	0.02
mlp_model	PhysChem	DRFP OOD Clusters - S.R.	Nat.C.24	0.43	0.0	0.46	0.01	0.38	0.0	0.51	0.01	0.51	0.02
knn_model	PhysChem	DRFP OOD Clusters - S.R.	Nat.C.24	0.46	0.01	0.49	0.01	0.5	0.02	0.48	0.03	0.51	0.02
gaussianNB_model	PhysChem	DRFP OOD Clusters - S.R.	Nat.C.24	0.34	0.01	0.38	0.01	0.37	0.0	0.39	0.01	0.41	0.02
rf_model	PhysChem	DRFP OOD Clusters - S.R.	Chem.S.23	0.59	0.02	0.51	0.02	0.13	0.16	0.69	0.02	0.52	0.02
gradient_boosting_model	PhysChem	DRFP OOD Clusters - S.R.	Chem.S.23	0.45	0.01	0.52	0.01	0.62	0.01	0.2	0.02	0.42	0.01
lr_model	PhysChem	DRFP OOD Clusters - S.R.	Chem.S.23	0.43	0.01	0.47	0.01	0.43	0.02	0.51	0.01	0.41	0.01
mlp_model	PhysChem	DRFP OOD Clusters - S.R.	Chem.S.23	0.43	0.04	0.46	0.03	0.38	0.08	0.53	0.03	0.51	0.05
knn_model	PhysChem	DRFP OOD Clusters - S.R.	Chem.S.23	0.42	0.01	0.5	0.0	0.59	0.01	0.25	0.02	0.44	0.01
gaussianNB_model	PhysChem	DRFP OOD Clusters - S.R.	Chem.S.23	0.35	0.01	0.4	0.01	0.36	0.01	0.44	0.01	0.37	0.01
rf_model	PhysChem	DRFP OOD Clusters - S.R.	Sci.23	0.4	0.01	0.5	0.0	0.0	0.0	0.71	0.01	0.38	0.01
gradient_boosting_model	PhysChem	DRFP OOD Clusters - S.R.	Sci.23	0.54	0.01	0.54	0.01	0.46	0.01	0.61	0.01	0.48	0.01
lr_model	PhysChem	DRFP OOD Clusters - S.R.	Sci.23	0.47	0.01	0.46	0.01	0.35	0.01	0.56	0.01	0.44	0.0
mlp_model	PhysChem	DRFP OOD Clusters - S.R.	Sci.23	0.41	0.01	0.45	0.0	0.27	0.02	0.59	0.01	0.43	0.02
knn_model	PhysChem	DRFP OOD Clusters - S.R.	Sci.23	0.46	0.0	0.47	0.0	0.23	0.01	0.63	0.0	0.41	0.01
gaussianNB_model	PhysChem	DRFP OOD Clusters - S.R.	Sci.23	0.44	0.01	0.5	0.0	0.09	0.0	0.7	0.01	0.42	0.02
rf_model	PhysChem	DRFP OOD Clusters - S.R.	JnJ25	0.61	0.01	0.58	0.02	0.61	0.04	0.55	0.06	0.62	0.02
gradient_boosting_model	PhysChem	DRFP OOD Clusters - S.R.	JnJ25	0.61	0.01	0.6	0.01	0.68	0.04	0.5	0.04	0.61	0.01
lr_model	PhysChem	DRFP OOD Clusters - S.R.	JnJ25	0.56	0.01	0.54	0.01	0.59	0.03	0.49	0.05	0.55	0.01
mlp_model	PhysChem	DRFP OOD Clusters - S.R.	JnJ25	0.6	0.02	0.58	0.01	0.63	0.03	0.54	0.01	0.65	0.03
knn_model	PhysChem	DRFP OOD Clusters - S.R.	JnJ25	0.52	0.01	0.5	0.01	0.4	0.02	0.57	0.02	0.52	0.02
gaussianNB_model	PhysChem	DRFP OOD Clusters - S.R.	JnJ25	0.63	0.02	0.62	0.01	0.65	0.04	0.57	0.03	0.63	0.01
rf_model	PhysChem	DRFP OOD Clusters - S.R.	JACS.25	0.43	0.02	0.5	0.0	0.61	0.0	0.02	0.02	0.38	0.01
gradient_boosting_model	PhysChem	DRFP OOD Clusters - S.R.	JACS.25	0.58	0.0	0.58	0.0	0.55	0.01	0.61	0.0	0.48	0.0
lr_model	PhysChem	DRFP OOD Clusters - S.R.	JACS.25	0.62	0.0	0.6	0.0	0.53	0.01	0.66	0.0	0.52	0.0
mlp_model	PhysChem	DRFP OOD Clusters - S.R.	JACS.25	0.56	0.0	0.56	0.0	0.55	0.0	0.53	0.0	0.6	0.01
knn_model	PhysChem	DRFP OOD Clusters - S.R.	JACS.25	0.58	0.0	0.58	0.0	0.58	0.0	0.56	0.0	0.51	0.0
gaussianNB_model	PhysChem	DRFP OOD Clusters - S.R.	JACS.25	0.63	0.0	0.59	0.0	0.59	0.0	0.57	0.0	0.55	0.0
rf_model	DRFP	DRFP OOD Clusters - S.R.	Sci.15	0.51	0.01	0.5	0.0	0.0	0.0	0.74	0.01	0.41	0.03
gradient_boosting_model	DRFP	DRFP OOD Clusters - S.R.	Sci.15	0.51	0.02	0.5	0.0	0.0	0.0	0.74	0.01	0.41	0.02
lr_model	DRFP	DRFP OOD Clusters - S.R.	Sci.15	0.5	0.01	0.5	0.0	0.0	0.0	0.74	0.01	0.42	0.01
mlp_model	DRFP	DRFP OOD Clusters - S.R.	Sci.15	0.45	0.01	0.5	0.0	0.01	0.0	0.74	0.01	0.39	0.01
knn_model	DRFP	DRFP OOD Clusters - S.R.	Sci.15	0.47	0.01	0.5	0.0	0.08	0.01	0.73	0.01	0.4	0.02
gaussianNB_model	DRFP	DRFP OOD Clusters - S.R.	Sci.15	0.5	0.0	0.5	0.0	0.0	0.0	0.74	0.01	0.71	0.01
rf_model	DRFP	DRFP OOD Clusters - S.R.	Chem.S.16	0.58	0.03	0.51	0.0	0.02	0.01	0.7	0.01	0.57	0.02
gradient_boosting_model	DRFP	DRFP OOD Clusters - S.R.	Chem.S.16	0.57	0.01	0.49	0.0	0.13	0.02	0.67	0.01	0.5	0.01
lr_model	DRFP	DRFP OOD Clusters - S.R.	Chem.S.16	0.49	0.0	0.5	0.0	0.17	0.0	0.66	0.01	0.46	0.01
mlp_model	DRFP	DRFP OOD Clusters - S.R.	Chem.S.16	0.52	0.0	0.52	0.0	0.23	0.0	0.67	0.01	0.51	0.01
knn_model	DRFP	DRFP OOD Clusters - S.R.	Chem.S.16	0.54	0.01	0.51	0.0	0.12	0.0	0.69	0.01	0.51	0.01
gaussianNB_model	DRFP	DRFP OOD Clusters - S.R.	Chem.S.16	0.5	0.0	0.5	0.0	0.04	0.0	0.69	0.01	0.51	0.01
rf_model	DRFP	DRFP OOD Clusters - S.R.	Sci.18	0.47	0.01	0.5	0.0	0.56	0.0	0.02	0.01	0.37	0.01
gradient_boosting_model	DRFP	DRFP OOD Clusters - S.R.	Sci.18	0.46	0.01	0.51	0.0	0.56	0.0	0.13	0.0	0.35	0.01
lr_model	DRFP	DRFP OOD Clusters - S.R.	Sci.18	0.5	0.0	0.5	0.0	0.56	0.0	0.06	0.0	0.39	0.0
mlp_model	DRFP	DRFP OOD Clusters - S.R.	Sci.18	0.48	0.0	0.5	0.0	0.55	0.0	0.04	0.0	0.37	0.0
knn_model	DRFP	DRFP OOD Clusters - S.R.	Sci.18	0.5	0.01	0.5	0.01	0.53	0.01	0.26	0.08	0.48	0.02
gaussianNB_model	DRFP	DRFP OOD Clusters - S.R.	Sci.18	0.5	0.0	0.5	0.0	0.53	0.0	0.26	0.0	0.59	0.0
rf_model	DRFP	DRFP OOD Clusters - S.R.	Nat.C.24	0.57	0.01	0.5	0.0	0.0	0.0	0.66	0.01	0.55	0.01
gradient_boosting_model	DRFP	DRFP OOD Clusters - S.R.	Nat.C.24	0.55	0.01	0.51	0.0	0.18	0.0	0.65	0.01	0.54	0.02
lr_model	DRFP	DRFP OOD Clusters - S.R.	Nat.C.24	0.58	0.0	0.57	0.0	0.53	0.01	0.6	0.01	0.57	0.01
mlp_model	DRFP	DRFP OOD Clusters - S.R.	Nat.C.24	0.53	0.0	0.52	0.0	0.56	0.01	0.45	0.0	0.63	0.01
knn_model	DRFP	DRFP OOD Clusters - S.R.	Nat.C.24	0.45	0.0	0.5	0.0	0.1	0.01	0.65	0.01	0.47	0.02
gaussianNB_model	DRFP	DRFP OOD Clusters - S.R.	Nat.C.24	0.53	0.0	0.53	0.0	0.6	0.01	0.41	0.01	0.69	0.01
rf_model	DRFP	DRFP OOD Clusters - S.R.	Chem.S.23	0.46	0.01	0.48	0.01	0.54	0.05	0.32	0.08	0.43	0.01
gradient_boosting_model	DRFP	DRFP OOD Clusters - S.R.	Chem.S.23	0.53	0.01	0.53	0.01	0.52	0.02	0.51	0.01	0.47	0.01
lr_model	DRFP	DRFP OOD Clusters - S.R.	Chem.S.23	0.41	0.01	0.43	0.0	0.42	0.01	0.45	0.01	0.39	0.01
mlp_model	DRFP	DRFP OOD Clusters - S.R.	Chem.S.23	0.38	0.01	0.46	0.0	0.14	0.01	0.63	0.0	0.41	0.01
knn_model	DRFP	DRFP OOD Clusters - S.R.	Chem.S.23	0.44	0.0	0.49	0.0	0.6	0.01	0.12	0.0	0.51	0.0
gaussianNB_model	DRFP	DRFP OOD Clusters - S.R.	Chem.S.23	0.5	0.0	0.5	0.0	0.62	0.01	0.02	0.01	0.72	0.0
rf_model	DRFP	DRFP OOD Clusters - S.R.	Sci.23	0.59	0.01	0.6	0.0	0.44	0.01	0.71	0.01	0.56	0.01
gradient_boosting_model	DRFP	DRFP OOD Clusters - S.R.	Sci.23	0.59	0.0	0.57	0.0	0.45	0.01	0.68	0.01	0.57	0.01
lr_model	DRFP	DRFP OOD Clusters - S.R.	Sci.23	0.48	0.01	0.5	0.01	0.42	0.0	0.57	0.01	0.43	0.01
mlp_model	DRFP	DRFP OOD Clusters - S.R.	Sci.23	0.53	0.01	0.53	0.0	0.44	0.0	0.62	0.0	0.55	0.01
knn_model	DRFP	DRFP OOD Clusters - S.R.	Sci.23	0.51	0.0	0.5	0.0	0.57	0.01	0.3	0.0	0.58	0.01
gaussianNB_model	DRFP	DRFP OOD Clusters - S.R.	Sci.23	0.49	0.0	0.49	0.0	0.05	0.01	0.7	0.01	0.41	0.01
rf_model	DRFP	DRFP OOD Clusters - S.R.	JnJ25	0.63	0.02	0.5	0.0	0.0	0.0	0.66	0.01	0.64	0.02
gradient_boosting_model	DRFP	DRFP OOD Clusters - S.R.	JnJ25	0.61	0.02	0.54	0.01	0.38	0.02	0.63	0.01	0.58	0.04
lr_model	DRFP	DRFP OOD Clusters - S.R.	JnJ25	0.51	0.01	0.5	0.01	0.46	0.0	0.54	0.02	0.51	0.01
mlp_model	DRFP	DRFP OOD Clusters - S.R.	JnJ25	0.61	0.01	0.58	0.01	0.58	0.02	0.59	0.01	0.67	0.01
knn_model	DRFP	DRFP OOD Clusters - S.R.	JnJ25	0.65	0.01	0.62	0.01	0.64	0.02	0.6	0.02	0.72	0.02
gaussianNB_model	DRFP	DRFP OOD Clusters - S.R.	JnJ25	0.53	0.01	0.53	0.0	0.59	0.01	0.46	0.02	0.68	0.01
rf_model	DRFP	DRFP OOD Clusters - S.R.	JACS.25	0.51	0.01	0.5	0.0	0.0	0.0	0.72	0.0	0.44	0.01
gradient_boosting_model	DRFP	DRFP OOD Clusters - S.R.	JACS.25	0.46	0.0	0.48	0.0	0.12	0.0	0.68	0.0	0.4	0.0
lr_model	DRFP	DRFP OOD Clusters - S.R.	JACS.25	0.44	0.0	0.48	0.0	0.13	0.0	0.68	0.0	0.4	0.0
mlp_model	DRFP	DRFP OOD Clusters - S.R.	JACS.25	0.47	0.0	0.49	0.0	0.09	0.0	0.7	0.0	0.41	0.0
knn_model	DRFP	DRFP OOD Clusters - S.R.	JACS.25	0.44	0.0	0.45	0.0	0.38	0.0	0.52	0.0	0.42	0.0
gaussianNB_model	DRFP	DRFP OOD Clusters - S.R.	JACS.25	0.51	0.0	0.51	0.0	0.31	0.0	0.67	0.0	0.52	0.0

5 Supplementary Section 5 - Production Model Hyperparameters

5.1 Motivation

The benchmarking experiments presented in Section 4 deliberately employ near-default hyperparameters (Supplementary Table 3) that are held constant across all training corpora and feature sets. This design choice ensures that observed performance differences reflect the intrinsic informativeness of each data source rather than artifacts of model tuning. However, when training a final model for laboratory deployment, maximizing predictive performance becomes the priority. To this end, we performed a systematic grid search over hyperparameters for the three top-performing model families, Random Forest (RF), Histogram-based Gradient Boosting (HistGBT), and Multi-Layer Perceptron (MLP) evaluated under the same out-of-distribution (OOD) cluster protocol used throughout this work.

5.2 Grid Search Protocol

For RF, which is computationally inexpensive, we evaluated 12 hyperparameter combinations. For HistGBT and MLP, which require substantially more training time on high-dimensional features, we evaluated 6 combinations each (Supplementary Table 7). Each configuration was trained on the train clusters and evaluated on the held-out test clusters across 3 random cluster initializations, with K=12 clusters split 9 (train) / 3 (test). Performance was averaged over all iterations and splits, giving a robust estimate of OOD generalization per configuration.
Two feature representations were tested independently:

- **DRFP** (2048): Full-reaction Difference Reaction Fingerprint.
- **Split** (2069): Substrate-product DRFP (2048) concatenated with reagent-only QM descriptors (21: catalyst + base + solvent).

This yields a total of $(12+6+6) \times 2 = 48$ model–feature–hyperparameter configurations, each evaluated across ∼12 train/test splits (3 iterations × 4 cluster groups per iteration).

Supplementary Table 7: Hyperparameter grids explored for each model family.

Model	Parameter	Values	Fixed
Random Forest (12)	$\mathtt{n}_e stimators|$	200, 300, 500	$\mathtt{max}_d epth| = None, \mathtt{min}_s amples_s plit| = 4$
	$\mathtt{min}_s amples_l eaf|$	1, 3	criterion|=entropy, $\mathtt{class}_w eight| = balanced$
	$\mathtt{max}_f eatures|$	log2, sqrt	
HistGradientBoosting (6)	$\mathtt{max}_i ter|$	200, 400	$\mathtt{max}_d epth| = 6, \mathtt{random}_s tate| = 42$
	$\mathtt{learning}_r ate|$	0.05, 0.1, 0.2	
MLP (6)	$\mathtt{hidden}_l ayer_s izes|$	(64,16), (100,20), (200,50)	solver|=L-BFGS, $\mathtt{max}_i ter| = 300$
	alpha|	10^{-3}, 10^{-4}	

5.3 Results

Supplementary Table 8 summarizes the best hyperparameter configuration per model and feature set, ranked by mean OOD ROC AUC.

Supplementary Table 8: Best hyperparameter configuration per model and feature set, ranked by OOD ROC AUC. Results averaged over 3 cluster initializations (∼12 train/test splits).

Feature Set	Model	ROC AUC	± Std	Bal. Acc.	± Std	F1	± Std	Best Hyperparameters
Split	RF	**0.847**	0.054	**0.767**	0.062	**0.735**	0.089	$n_e st\| = 500$, leaf|=1, feat|=log2
Split	HGB	0.825	0.072	0.737	0.072	0.687	0.118	iter|=400, lr|=0.2
DRFP	RF	0.811	0.071	0.709	0.094	0.593	0.202	$n_e st\| = 300$, leaf|=1, feat|=log2
DRFP	HGB	0.808	0.079	0.718	0.072	0.638	0.139	iter|=200, lr|=0.1
Split	MLP	0.791	0.091	0.741	0.066	0.715	0.077	hidden|=(100,20), α=10^{-3}
DRFP	MLP	0.727	0.080	0.671	0.069	0.609	0.121	hidden|=(200,50), α=10^{-4}

The split feature representation (substrate-product DRFP + reagent QM) consistently outperformed full-reaction DRFP across all three model families. The performance gain from changing feature set was larger than the gap between models within the same feature set: RF improved from ROC AUC 0.811 to 0.847 when switching from DRFP to split features, whereas RF vs. HistGBT on split features differed by only 0.847 vs. 0.825. This suggests that when the feature representation cleanly encodes the relevant chemical information, separating substrate structural identity from reagent properties, a simple model that does not distort it is sufficient.

Among models, RF achieved the highest ROC AUC (0.847) and F1 (0.735) on split features. Notably, the split representation resolved a precision–recall imbalance observed with DRFP: RF on DRFP had high precision (0.824) but low recall (0.498), while on split features precision remained high (0.758) with recall improving substantially (0.722). The reagent QM descriptors provide the additional signal needed to identify positive reactions that the substrate-only fingerprint misses.

5.4 Model Selection Rationale

No single model dominates across all benchmarks,the best model varies by data source, feature set, and metric (for example, KNN + DRFP performed well in the data source benchmark on ROC AUC, while RF + split features wins in the production model hyperparameter optimization). That said, RF is consistently competitive, and several properties explain why simple models perform well in this setting.

In our view, the features are the most important aspect. As shown in Supplementary Table 8, the performance gain from changing feature representation is larger than the gap between models within the same feature set. This suggests that when the feature representation cleanly encodes the relevant chemical information, a simple model that does not distort it is sufficient.

RF is naturally suited to the feature structure of this task. The split features consist of 2048 sparse binary fingerprint bits concatenated with 21 continuous physicochemical descriptors. Tree-based splits on binary fingerprint bits directly correspond to “substructure present/absent” decisions, which is the underlying chemical question. RF’s natural subsampling means no individual tree can memorize training-distribution-specific patterns. The ensemble average retains only patterns that recur across many random subsets, which are more likely to be genuinely predictive rather than distribution artifacts.

We also tested BEE (BERT-Enriched Embeddings), a large pretrained transformer model operating on SMILES-based representations, a fundamentally different feature type. Performance for BEE was similar to the best ML models observed in the benchmarks. However, given the large number of train–test splits required by our OOD evaluation

protocol, the performance benefit of more complex models like BEE did not justify the increased computational cost and complexity for this single-reaction-type setting with at best ∼17,000 training samples per split. We expect the performance advantage of pre-trained transformers to become more substantial as additional reaction types are included and/or larger chemical spaces are covered in training and test sets.

5.5 Production Model

Based on the grid search results, RF with split features was selected as the production model, using the following hyperparameters: `n_estimators`=500, `min_samples_leaf`=1, `max_features`=log2. This configuration was retrained on the full dataset (27,497 reactions from all 8 sources) and saved as a serialized artifact for laboratory deployment. The artifact includes the fitted model, the fitted scaler, and metadata (hyperparameters, OOD metrics, training date) to ensure reproducibility. The production model achieves OOD performance of ROC AUC = 0.847, Balanced Accuracy = 0.767, and F1 = 0.735 on the held-out cluster evaluation.

5.6 Reagent Recommender Workflow

To deploy the production model as a reagent recommender, we implement the following automated pipeline. First, a *reagent dictionary* is constructed by selecting catalysts, bases, and solvents that appear above a user-defined frequency threshold in the training corpus, ensuring that the model has seen sufficient examples of each reagent to make informed predictions. For each reagent, the corresponding physicochemical descriptors used during training are stored alongside the reagent name and SMILES.

Given a target substrate pair (aryl halide and amine), the pipeline computes the substrate-product DRFP fingerprint (2048 bits) and then enumerates all possible combinations of catalysts, bases, and solvents from the reagent dictionary. For each combination, the substrate fingerprint is concatenated with the reagent physicochemical descriptors to form the 2069-dimensional split feature vector, which is then scored by the production model. The output is a continuous predicted probability of success (0–1) for every reagent combination, enabling the chemist to rank all candidates and select the most promising conditions for experimental testing.

The reagent recommender was developed to facilitate the rational design of high-throughput experimentation (HTE) plates. By aggregating and analyzing predictions across the entire reaction space for a given substrate pair, we can prioritize catalysts, bases, and solvents that are most likely to succeed. For instance, a 24-well plate HTE may be constructed by combining the top six catalysts with the top two bases and top two solvents. The size and composition of each array can be adjusted to meet specific experimental goals, and the final plate design is determined by the investigator, who integrates their domain expertise with the recommender's ranked suggestions.

Supplementary Figure 9 illustrates this workflow on an example substrate pair. The prediction distribution across all candidate reagent combinations is shown as a histogram, color-coded from red (low predicted probability) through white (borderline) to green (high predicted probability). From the ranked predictions, the top-scoring combinations are selected to populate a focused HTE plate, minimizing the number of experiments needed to identify viable reaction conditions. In conclusion, the reagent recommender was developed to facilitate the rational design of high-throughput experimentation (HTE)

plates. By aggregating and analyzing predictions across the entire reaction space for a given substrate pair, we can prioritize catalysts, bases, and solvents that are most likely to succeed. For instance, a 24-well plate HTE may be constructed by combining the top six catalysts with the top two bases and top two solvents. The size and composition of each array can be adjusted to meet specific experimental goals, and the final plate design is determined by the investigator, who integrates their domain expertise with the recommender's ranked suggestions. The full pipeline including reagent dictionary construction, feature computation, and plate design is provided as a self-contained notebook in the repository.

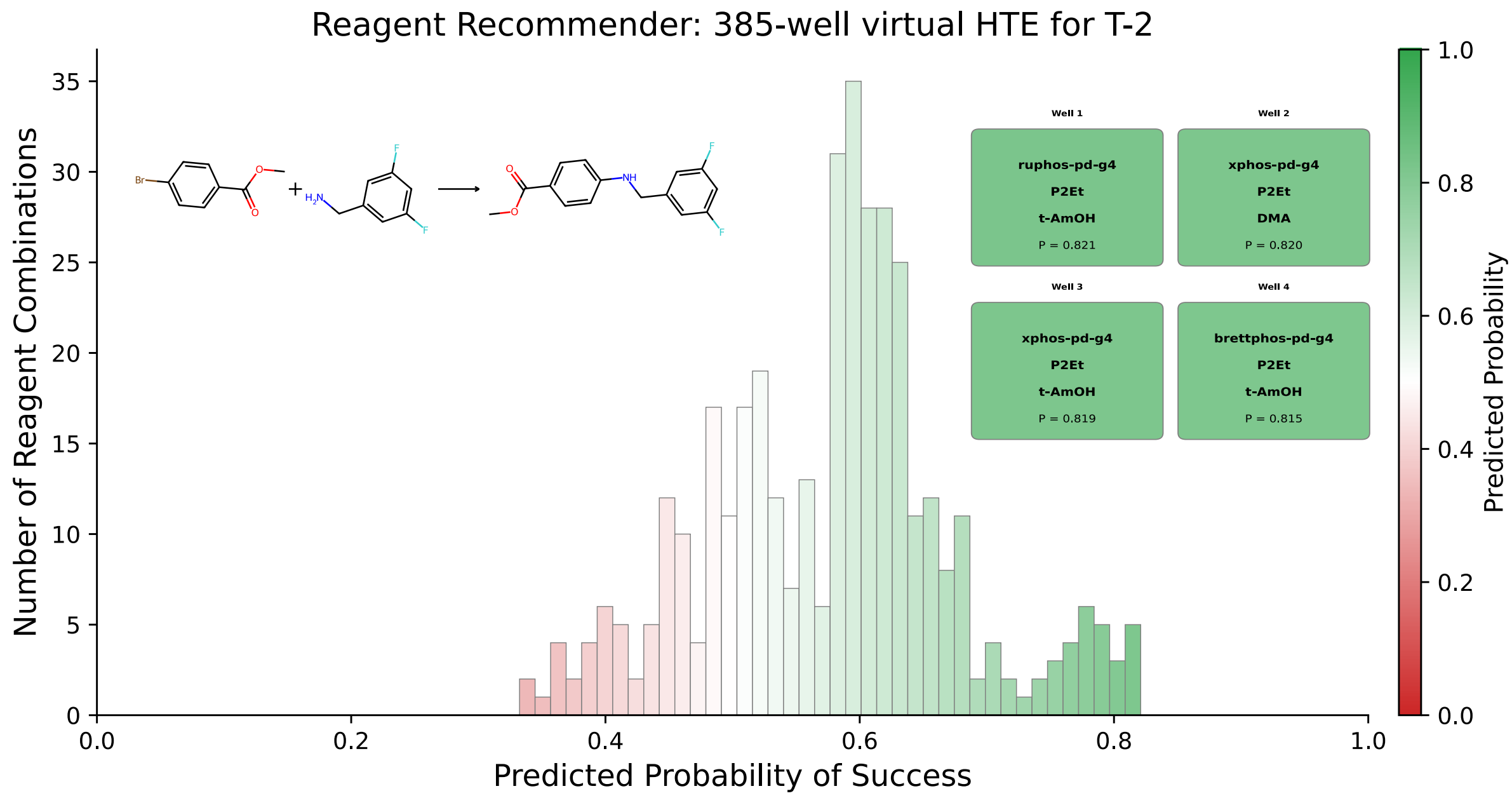

Supplementary Figure 9: Reagent recommender example. (a) Target substrate pair. (b) Distribution of predicted success probabilities across all candidate reagent combinations from the reagent dictionary. (c) Highest-ranked reagent combinations, as input for chemist.

Inventory of Source Data

This section provides an inventory of numerical source data files supplied to support figures in the main manuscript.

Figure 3 — Dataset Composition and CRDS vs OOD Performance

Figure 3a — Dataset composition statistics
Source data file: `Fig_3a.csv`
Contents: Raw dataset composition metrics for each training corpus used in CRDS calculation and OOD benchmarking. Columns include unique reactant pairs, unique reagent combinations, total reaction samples, and dataset name.

Figure 3b,c,d — CRDS values and correlation analysis
Source data files:

- `table2_dataset_composition_crds.csv` — Dataset composition and computed CRDS values for all training corpora
- `table3_hyperparameters_benchmark.csv` — Hyperparameter settings for benchmark models used in OOD performance evaluation

The CRDS correlation values shown in Figure 3 panels are computed from the best OOD performance metrics (Supplementary Table 4) combined with CRDS values from `table2_dataset_composition_crds.csv`.

Figure 5 — Experimental Validation Results

Figure 5a — Rescue of previously unsuccessful substrate pairs
Source data file: `BH_Manu_NewConditions_20250310_result.csv`
Location: `data/` directory in repository
Contents: Experimental validation results (118 reactions) for model-guided rescue of substrate pairs that previously failed under tested conditions. Columns include substrate SMILES, predicted reagent combinations (catalyst, base, solvent), model confidence scores, prior failure count, and measured yields.

Figure 5b — Prospective validation on unseen substrates
Source data file: `Case1_selections_20250408_result.csv`
Location: `data/` directory in repository
Contents: Prospective experimental validation results (300 reactions) for model predictions on unseen aryl halide–amine pairs not present in training data. Columns include substrate SMILES, predicted reagent combinations, model confidence scores, clustering assignments, and measured yields.
